# Retreatment rates and postprocedural complications are higher than expected after BPH surgeries: a US healthcare claims and utilization study

**DOI:** 10.1038/s41391-023-00741-8

**Published:** 2023-10-26

**Authors:** Steve Kaplan, Ronald P. Kaufman, Thomas Mueller, Dean Elterman, Bilal Chughtai, Daniel Rukstalis, Henry Woo, Claus Roehrborn

**Affiliations:** 1 Mount Sinai, New York, NY USA; 2 Albany Medical College, Albany, NY USA; 3 New Jersey Urology, Voorhees, NJ USA; 4 University Health Network, Toronto, ON Canada; 5 Weill Cornell Medical Center, New York, NY USA; 6 Prisma Health USC Medical Group, Columbia, SC USA; 7 University of Sydney, Sydney, Australia; 8 UT Southwestern Medical Center, Dallas, TX USA

**Keywords:** Outcomes research, Prostatic diseases

## Abstract

**Background:**

Up to 50% of men over 50 and 80% over 80 are affected by BPH. Shared decision-making regarding BPH treatment options can benefit from an improved understanding of relative risks and benefits for various treatments.

**Methods:**

Data for this longitudinal retrospective population-based cohort study were obtained from a random sample of US Medicare and commercial claims (IBM Watson MarketScan) and restricted to men undergoing BPH surgery (TURP, PVP, PUL, WVTT) from 2015 to 2021 across all sites of service. Retreatments included Holmium laser enucleation and index procedures. Main outcomes were rates of retreatment and procedural complications over 1 year, identified via CPT and ICD-9/10CM codes. Procedural complications that occurred at least 1 day post-index treatment were assessed, as were surgical retreatments with patients who had at least 1 and 5 year’s-worth of data. Baseline phenotype characterization did not control for symptomatology and was limited to age, comorbidities, and BMI. Univariate cumulative incidence estimates, cumulative proportion and log-rank tests justified inclusion for covariate (e.g., age, comorbidities) adjustment in Cox proportional hazard models.

**Results:**

43,147 men diagnosed with BPH underwent 22,629 TURP, 11,392 PVP, 7,529 PUL, and 1,597 WVTT. At 1-year post-index: PUL was associated with the lowest rate of complication (PUL 15%, TURP 17%; PVP 19%, ; WVTT 26%); retreatment rates were not different (TURP 5.3%, PVP 5.3%, PUL 5.9%, WVTT 6.2%). At 5 years post-index: retreatment was lowest for TURP (7.0%) and was not significantly different between PVP and PUL (8.9% and 11.6%, respectively).

**Conclusions:**

Real-world patients diagnosed with BPH may be selected to undergo one of the various available therapies based on patient preference or baseline phenotype. These therapies, however, are associated with different risks for complications. The results of this study suggest that within one year of BPH surgery, one-in-twenty patients may require retreatment regardless of treatment choice, and for some technologies as many as one-in-four may require treatment for a complication.

## Introduction

Benign prostatic hyperplasia (BPH) associated with lower urinary tract symptoms (LUTS) is an age-related healthcare issue involving millions of men and costing billions of dollars each year in North America [1]. If left untreated, BPH can affect patients’ quality of life through loss of sleep, reduced productivity, and impaired sex life [2–4]. Up to 70% of patients managed on medication may discontinue usage [5] due to lack of efficacy and/or intolerable side effects, yet only a small fraction of patients select traditional surgery such as transurethral resection of the prostate (TURP) and photoselective vaporization of the prostate (PVP) [6]. The American Urological Association (AUA) states that minimally invasive surgical therapies (MIST), i.e., Prostatic Urethral Lift (PUL) and water vapor thermal therapy (WVTT) [7], should be considered as treatment options for BPH patients, as they can alleviate symptoms with faster recovery and fewer permanent side effects [8].

Rates of surgical retreatment, complications, and adverse events (AEs) are important considerations when assessing BPH therapies. However, comparing different randomized controlled trials (RCTs) for these outcomes can be limiting owing to varying methodologies. Recent attempts to compare BPH treatments utilized cost-effectiveness statistical models driven by RCT inputs; [9, 10] however, these analyses assumed equitability across clinical studies without a demonstration of homogeneity, and the RCT inputs may not be reflective of real-life practice.

In an attempt to overcome limitations of RCT-based comparisons, we analyzed US Medicare and commercial insurance claims data to report real-world rates and risks of surgical retreatment and procedural complications following treatment with MIST (PUL and WVTT) or traditional surgery (TURP and PVP). This patient-level, longitudinal, observational analysis uses a standard definition for procedural complications and surgical retreatment across the treatment modalities. Like comparisons of technologies based only on published data from RCTs, this analysis approach offers limited control over differences in baseline characteristics. The standardization of retreatment and complication definitions, however, is a unique benefit of this type of real-world analysis which is not possible when trying to compare disparately conducted published RCTs.

## Methods

### BPH index procedures

PUL using the UroLift System (Teleflex Inc., Pennsylvania, USA) reduces prostatic obstruction via permanent UroLift implants [11, 12]. WVTT using the Rezūm System (Boston Scientific, Marlborough, Massachusetts, USA), causes prostatic cell death via water vapor injection [13]. PVP with GreenLight Laser (Boston Scientific, Marlborough, Massachusetts, USA) vaporizes prostate tissue. Transurethral resection of the prostate (TURP) cuts and removes sections of the prostate via resectoscope.

### Claims data

A random representative sample ( ~ 10% of all US BPH claims) was acquired from IBM Watson Health Marketscan Research, a nationwide database with de-identified, individual-level claims from outpatient, inpatient, and prescription drug services for >230 million privately insured patients in the US. Payers included Medicare Administrative Contractors and commercial insurance.

### Study population & definitions

The study population included males with LUTS secondary to BPH from January 2015-June 2021; WVTT analysis began after FDA clearance (August 27, 2015). BPH patients were identified via International Classification of Diseases Clinical Modifications, 9th & 10th additions (ICD-9/10 CM) (Supplementary Table 1). The top four index procedures performed at the time of analysis were TURP, PVP, PUL, and WVTT. As only 92 holmium laser enucleation (HoLEP) procedures were identified as outpatient index procedures in this dataset, HoLEP was not included but was identified as a possible retreatment procedure along with the aforementioned index procedures. Index procedures and surgical retreatments were identified using Current Procedural Terminology (CPT) or ICD-10 procedural coding system (PCS) (Supplementary Table 2). WVTT procedures performed using TUNA codes were excluded from this analysis. Procedural complications, i.e., complications requiring a return procedure in the outpatient setting ≥1d after the index procedure, were identified utilizing CPT, ICD-10 PCS, and ICD 9/10 CM codes (Supplementary Table 3). Adverse events were defined as ICD-9/10 CM diagnostic codes newly captured with procedural complications and/or surgical retreatments. All codes were deemed relevant (i.e., comprehensive) following BPH surgery by contributing authors (listed in Supplementary Tables 1–3).

### Statistical analysis

#### Descriptive statistics

Statistical analysis utilized SAS v9.4 (Cary, North Carolina). Descriptive statistics were presented for baseline demographic and characteristic variables. Percentages were based on the number of non-missing observations.

#### Univariate analysis

Univariate cumulative incidence estimates were plotted for time to first surgical retreatment, procedural complication or AE through 365d post-index. Cumulative and specific proportion and log-rank test p-values were calculated. Univariate analysis was performed to justify inclusion of covariates in final Cox proportional hazard models.

#### Multivariate cox proportional hazard models

Cox Regression determined predictors of procedural complications and surgical retreatment. All models were stratified by treatment; covariates included cost of index procedure, age at time of index procedure, comorbidities, and AEs listed at the time of procedural complication/surgical retreatment.

Hazard Ratios and corresponding 95% confidence intervals were derived for all covariates.

## Results

**Fig. 1:**
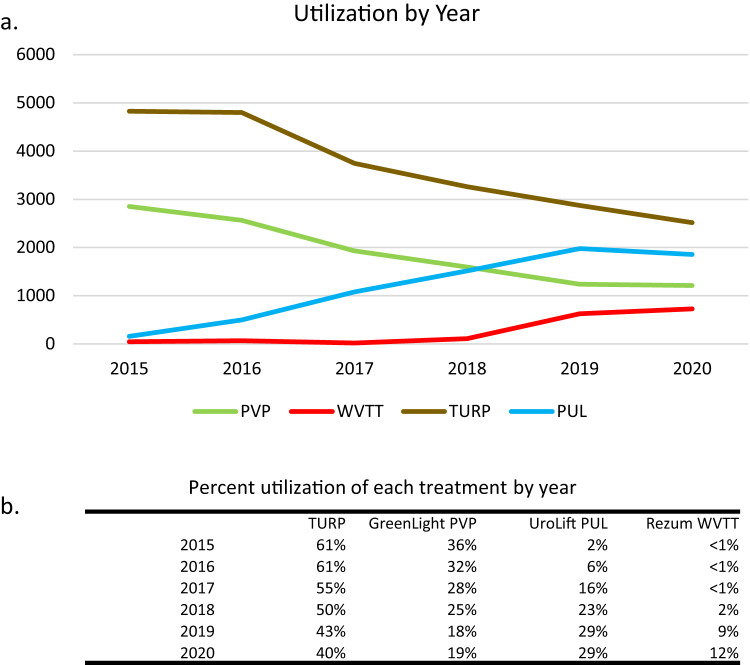
Utilization of select procedures by year. **a** Utilization numbers for each procedure by year (2015–2020); **b** Percent utilization of each treatment by year (of each year’s total) (2015–2020).

We identified 22,629 TURP, 11,392 PVP, 7,529 PUL, and 1,597 WVTT patients who underwent index procedures between January 2015 – June 2021. Data did not extend fully through 2021, therefore utilization was only evaluated through 2020. As utilization of MISTs (PUL and WVTT) increased from 2015-2020, utilization of traditional surgeries (TURP and PVP) declined. TURP utilization decreased from 61% in 2015 to 40% in 2020, and PVP decreased from 36% to 19% (Fig. 1). In 2015, utilization of PUL and WVTT was low (2% and 0.6%, respectively), but increased to 29% and 12%, respectively, in 2020 (Fig. 1).

Average baseline age was lower for PUL (64.4 y) and WVTT (63.6 y) vs. TURP and PVP (both 66.9 y) (Table 1). Overall rates of comorbidities were low, and most were not significantly different between treatment groups (Supplementary Table 4). Number of patient-years of follow-up ranged from 336 for WVTT to 48,055 for TURP (Table 2). Therefore, retreatment analyses were restricted to patients with ≥12 months of follow-up.

**Table 1:** Baseline demographics and average follow-up time of each procedure.

	TURP	GreenLight PVP	UroLift PUL	Rezum WVTT
n	22,629	11,392	7,529	1,585
Age	66.9	66.9	64.4	63.6
Follow-up time (years)	2.12	2.24	0.76	0.21
Patient Years	48,055	25,570	5,711	336

**Table 2:** Proportion of 1-year retreatment and 1-year complications by procedure.

	TURP	GreenLight PVP	UroLift PUL	Rezum WVTT	Log Rank p-value
**Proportion of Surgical Retreatment (1** **yr, ≥12mo follow-up)**	**5.3%**	**5.3%**	**5.9%**	**6.2%**	**0.2**
*Average time to first retreatment*	*29.8d*	*29.9d*	*29.9d*	*30.0d*	
**Proportion of any Procedural Complication (1** **yr)**	**17%**	**19%**	**15%**	**26%**	**<0.0001**
*Average time to first complication*	*35.1d*	*38.6d*	*57.7d*	*28.0d*	
*Retention or outflow-related complications*					
Bladder Irrigation	7.1%	6.9%	6.6%	11.9%	<0.0001
51700	“	“	“	“	
Catheterization	2.0%	2.3%	2.1%	6.5%	<0.0001
*Total n catheterizations*	*n* = 440	*n* = 254	n = 157	n = 86	
Self-catheterization					
51701	*n* = 56	*n* = 30	*n* = 13	*n* = 17	
Indwelling catheterization					
51702	*n* = 256	*n* = 146	*n* = 120	*n* = 55	
51703	*n* = 34	*n* = 29	*n* = 15	*n* = 14	
51102	*n* = 94	*n* = 49	*n* = 9	*n* = 0	
Urethral Stent Placement (Spanner)	0.00%	0.00%	0.04%	0.45%	<0.0001
*Cystoscopy-related complications*					
Cystoscopy	3.0%	3.6%	4.2%	3.8%	<0.0001
*Urethral repair/stricture/dilation*	1.2%	0.7%	0.4%	0.3%	<0.0001
*Bleeding-related complications*					
Fulguration	0.6%	0.4%	0.5%	0.5%	0.2
Bleeding Control	0.02%	0.9%	0.03%	0.0%	<0.0001
Clot Removal	0.4%	0.2%	0.09%	0.2%	<0.0001
*Other*					
Removal of Foreign Body, Calculus or Stent	0.4%	0.3%	0.2%	0.8%	0.004
Incontinence	0.0%	0.02%	0.03%	0.0%	0.1
Bladder Neck Contracture/Dilation/Repairs	0.03%	0.05%	0.11%	0.00%	0.08

Complications with <0.01% incidence for each treatment: transfusion, incontinence, infection.

### Procedural complications

**Fig. 2:**
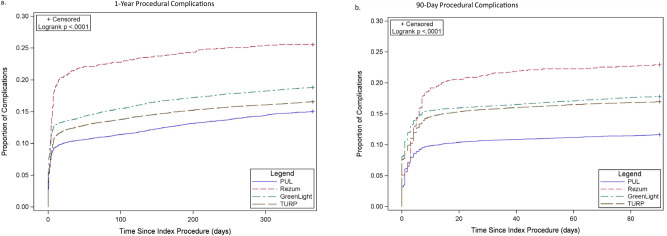
Procedural complications: cumulative incidence curve. **a** p < 0.0001 indicating significant difference through 1year, **b** *p* < 0.0001 indicating significant difference through 90 days.

Procedural complications at 365d post-index were lowest following PUL (15%) and highest following WVTT (26%) (p < 0.0001) (Table 2, Fig. 2a); the average time to first complication was longest for PUL (Table 2). Most complications occurred within ≤90d (Fig. 2b). Results were similar for Medicare-only patients.

The most common procedural complications after any index surgery were bladder irrigation, catheterization, and cystoscopy (Table 2, CPT code frequencies in Supplementary Table 5). Bladder irrigation was most common after WVTT, occurring in 11.9% of patients with a 13d mean onset time (PUL: 6.6%/40d, PVP: 6.9%/39d, and TURP: 7.1%/45d). Catheterization was also more common after WVTT procedures (WVTT: 6.5%/14d, TURP: 2.8%/104d, PVP: 2.7%/127d, PUL: 2.6%/71d) – these primarily consisted of new Foley catheters placed ≥1d post-procedure (Supplementary Table 5) and did not include immediate postoperative catheters or catheter removals. Cystoscopy rates were slightly higher after PUL (PUL 4.2%/357d, WVTT 3.8%/194d, TURP 3.0%/451d, PVP 3.6%/435d). Recorded infection rates were overall low.

### Adverse events

AEs in this analysis were defined as diagnostic codes newly captured at the time of an outpatient procedure for complications or surgical retreatment through 365d post-index procedure. Rates of AEs were generally low and not different between groups.

### Surgical retreatment

#### 1-year retreatment rates

**Fig. 3:**
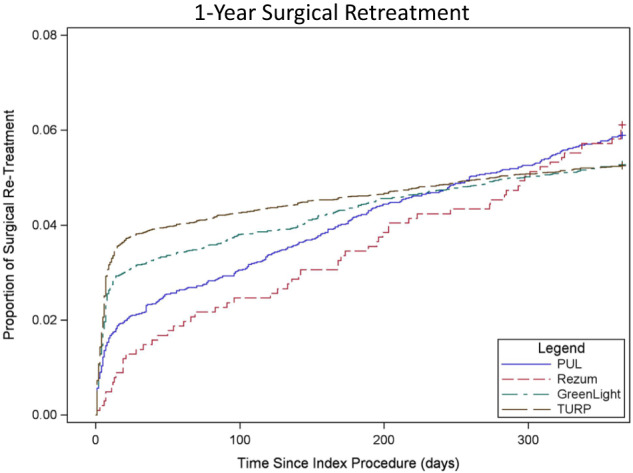
Surgical retreatment: cumulative incidence curve. p-0.2 indicating no difference through 1 year.

Rates of BPH surgery (TURP, PVP, PUL, WVTT, or HoLEP) occurring secondarily to the index procedure were similar among all procedures (TURP 5.3%, PVP 5.3%, PUL 5.9%, and WVTT 6.2%) (Table 2, Fig. 3). A notable proportion of TURP and PVP patients underwent surgical retreatment within 30 days (TURP 3.8%, PVP 3.2%), with sharp inclines early in the 30d post-op period, whereas MISTs gradually increased throughout 365d. WVTT and PUL retreatment rates were similar between Medicare and commercial; TURP and PVP commercial rates were slightly higher than Medicare (i.e., TURP-commercial 6% vs. TURP-Medicare 5.3%; and PVP-commercial 6% vs. PVP-Medicare 5.3%). Most surgical retreatments were performed using the original modality (Table 3a, b).

**Table 3:** 1- and 5-year surgical retreatment stratified by retreatment code. A: 1-Year Retreatment, B: 5-Year Retreatment.

3a. 1-Year Retreatment	TURP index	GreenLight PVP index	Rezum WVTT index	UroLift PUL index	Log Rank p-value
Total Patient #	20,341	10,533	1,051	5,802	
**Total Retreatment (%)**	**5.3% (1068)**	**5.3% (555)**	**6.2% (65)**	**5.9% (342)**	**0.2**
**PVP**	**4.5% (49)**	**61.6% (342)**	**7.7% (5)**	**9.1% (31)**	**<0.0001**
52648	0.24% (49, 100%)	3.2% (342, 100%)	0.48% (5, 100%)	0.53% (31, 100%)	<0.0001
**HoLEP**	**0.3% (3)**	**0.7% (4)**	**0.00% (0)**	**0.9% (3)**	**0.4**
52649	0.01% (3, 100%)	0.04% (4, 100%)	0.00% (0)	0.05% (3, 100%)	0.4
**PUL**	**0.6% (6)**	**0.3% (2)**	**10.8% (7)**	**52% (178)**	**<0.0001**
52441	0.02% (5, 83%)	0.01% (1, 50%)	0.60% (6, 86%)	2.65% (155, 87%)	<0.0001
C9739	0.00% (0)	0.00% (0)	0.00% (0)	0.09% (5, 3%)	<0.0001
C9740	0.00% (1, 17%)	0.01% (1, 50%)	0.10% (1, 14%)	0.31% (18, 10%)	<0.0001
**WVTT**	**<0.01% (1)**	**0.3% (2)**	**41.5% (27)**	**1.5% (5)**	**<0.0001**
53899	0.00% (0)	0.01% (1, 50%)	2.1% (22, 81%)	0.05% (3, 60%)	<0.0001
53854	0.00% (1, 100%)	0.01% (1, 50%)	0.48% (5, 19%)	0.03% (2, 40%)	<0.0001
**TURP**	**94.5% (1009)**	**36.9% (205)**	**40% (26)**	**36.5% (125)**	**<0.0001**
52500	0.05% (10, 1%)	0.06% (6, 3%)	0.10% (1, 4%)	0.00% (0)	0.3
52640	0.03% (6, 1%)	0.02% (2, 1%)	0.00% (0)	0.02% (1, 1%	0.9
52601	4.2% (845, 84%)	1.4% (148, 72%)	2.0% (21, 81%)	2.0% (118, 94%)	<0.0001
52630	0.73% (148, 15%)	0.47% (49, 24%)	0.38% (4, 15%)	0.10% (6, 5%)	<0.0001

3b. 5-Year Retreatment			
	TURP index	GreenLight PVP index	UroLift PUL index
Total Patient #	6,748	3,922	293
**Total Retreatment (%)**	**7.0% (476)**	**8.9% (348)**	**11.6% (34)**
**PVP**	**0.6% (40)**	**4.9% (193)**	**1.4% (4)**
52648	0.6% (40, 100.0%)	4.9% (40, 100.0%)	1.4% (4, 100.0%)
**HoLEP**	**0.0% (0)**	**0.0% (0)**	**0.0% (0)**
52649	0.0% (0, 0.0%)	0.0% (0, 0.0%)	0.0% (0, 0.0%)
**PUL**	**0.1% (7)**	**0.3% (11)**	**5.5% (16)**
52441	0.0% (2, 28.6%)	0.2% (7, 63.6%)	4.4% (13, 81.3%)
C9739	0.0% (2, 28.6%)	0.0% (1, 9.1%)	0.7% (2, 12.5%)
C9740	0.0% (3, 42.9%)	0.1% (3, 27.3%)	0.3% (1, 6.3%)
**WVTT**	**0.0% (1)**	**0.0% (0)**	**0.0% (0)**
53899	0.0% (1, 100%)	0.0% (0, 0.0%)	0.0% (0, 0.0%)
53854	0.0% (0, 0.0%)	0.0% (0, 0.0%)	0.0% (0, 0.0%)
**TURP**	**6.3% (428)**	**3.7% (144)**	**4.8% (14)**
52500	0.0% (0, 0.0%)	0.0% (0, 0.0%)	0.0% (0, 0.0%)
52640	0.0% (2, 0.5%)	0.0% (1, 0.7%)	0.0% (0, 0.0%)
52601	4.8% (327, 76.4%)	2.4% (93, 64.6%)	4.4% (13, 92.9%)
52630	1.4% (96, 22.4%)	1.1% (45, 31.3%)	0.0% (0, 0.0%)

#### 5-year retreatment rates

Although this dataset spans from 2015–2021, short average follow-up time prohibited analysis of WVTT analysis through five years. When data were restricted to patients with ≥60 months of follow-up (n = 6,748 TURP, 3,922 PVP, 293 PUL), 5-year retreatment was lowest for TURP (7.0%) and not significantly different between PVP and PUL (8.9% and 11.6%) (Table 3b). Less than 1% of PUL patients went on to repeated PUL procedures.

### Cox regression

Statistical models accounted for clinical variables such as age, comorbidities, site of service, and AEs, and assessed relative risks of procedural complications or surgical retreatment. PUL was associated with the lowest hazard for procedural complications at 365d (Table 4). Relative risks for complications vs. PUL were 23%, 33%, and 63% greater for TURP, PVP, and WVTT, respectively. The risk of surgical retreatment did not differ between procedures (Table 4). The source of insurance claims (Medicare vs. commercial) did not affect risk of complication or retreatment.

**Table 4:** Multivariate hazard analysis for 1-year procedural complication and 1-year retreatment (relative to PUL).

	Hazard Ratio	Chi-Square
**1-year Risk of Procedural Complication**		
***Treatment Variables***		
Rezum WVTT vs UroLift PUL	1.58	<0.0001
GreenLight PVP vs UroLift PUL	1.33	<0.0001
TURP vs UroLift PUL	1.23	<0.0001
**1-year Risk of Surgical Retreatment**		
***Treatment Variables***		
Rezum WVTT vs UroLift PUL	1.07	0.7
GreenLight PVP vs UroLift PUL	0.96	0.5
TURP vs UroLift PUL	0.95	0.5

## Discussion

This is the largest longitudinal healthcare claims study evaluating complication and retreatment rates after BPH treatment with TURP, PVP, PUL, and WVTT and the only study to include all outpatient sites of service. We observed higher 1-year retreatment rates than those reported in RCTs and prior publications. Here, TURP and PVP 1-year retreatment rates were both 5.3%, compared to 1–2% for TURP [11, 12] and 1.5% for PVP in prior publications [14, 15]. The 1-year retreatment rates were 5.9% for PUL and 6.2% for WVTT, compared to 5.0% [13, 16] and 2.2% in their respective RCTs.

The rate of procedural complications was lowest after PUL, and surgical retreatment occurred at similar rates after all procedures. Multivariate analyses adjusting for clinical variables found that the hazard of encountering a complication was lowest after PUL and was similar for 1-year retreatment among all procedures.

Complications related to urinary retention (i.e., bladder catheterization and irrigation) occurred more frequently than any other event. The data did not indicate whether these episodes were triggered by increased residual urine, total inability to urinate, or some other presentation. Furthermore, the code for bladder irrigation may refer to a “fill-and-pull” (voiding trial) or a more complex procedure (e.g., treating clots in the bladder). These bladder irrigations occurring in 11.9% of WVTT patients are new procedures that occur with a 13d post-index mean onset time. Though there is a possibility that some of these may be de-catheterizations, PVP and TURP, and indeed even PUL, would also be undergoing de-catheterization processes. PVP, TURP, and PUL would theoretically have shorter immediate post-index catheterization durations, yet the mean onset times of bladder irrigation for these procedures occurs at 39d, 45d, and 40d post-index, respectively. More telling is that corroborative data of new catheterization events occurring in 6.5% of WVTT patients with a mean onset time of 14d post-index; as average time to catheter removal in controlled studies was 3–5 days, these results suggest that the outflow-related complications may be secondary retention events.

Many TURP and PVP retreatments occurred ≤30d after the initial procedure, possibly representing procedures to treat bleeding – indeed, description of primary CPT codes for TURP and PVP include “control of postoperative bleeding.” Regarding WVTT retreatment, given its recent introduction, smaller cohort, and utilization of a non-specific CPT, we did not extrapolate outcomes beyond 1 year. Patients with ≥5 years of follow-up demonstrated lower retreatment rates with TURP vs PUL and PVP, suggesting superior long-term durability with TURP. There was no significant difference at 5 years between retreatment rates of PVP (8.9%) and PUL (11.6%), though lower patient numbers for PUL may attribute to lack of statistical significance. The 11.6% PUL 5-year retreatment rate in this database corroborates the 13.6% retreatment rate in the RCT LIFT trial in a real-world population, roughly twice the size.

Although the number of WVTT patients is much lower than that of TURP, these proportions reflect real-world utilization throughout 2015–2021. Moreover, WVTT acquired specific CPT/HCPCS codes in 2019; >90% of WVTT index procedures in this analysis utilized the new, specific CPT/HCPCS codes. Prior to this, procedures for WVTT were performed using an “unlisted procedure” code (53899) paired with a BPH diagnosis code [17]. We intentionally excluded the transurethral needle ablation (TUNA) CPT code (53852), which was also used in the early years of WVTT. This conservative approach may undercount WVTT procedures but provides the highest accuracy, given the heterogeneity of these claims. As HoLEP was not among the most common procedures at the time of analysis and small sample size may have introduced sampling bias, we did not assess downstream events post-HoLEP. However, retreatment rates for the top four index procedures in this analysis accounted for secondary HoLEP procedures.

Our study has several limitations. Insurance datasets do not provide patient level detail for symptom severity, prostate volume, etc. Although multivariate modeling adjusts for some baseline characteristics such as age and comorbidities, patient selection based on symptom severity or prostate volume may affect outcomes. Real-world analyses contain patient populations not commonly included in clinical trials. Indeed, in a recent real-world study conducted on patients from USA and Australia using PUL, the real-world population was older, had lower baseline IPSS, lower QoL, and higher Qmax than the LIFT RCT for PUL [18]. Although these variations in population between the real-world and clinical studies may alter results, real-world studies provide insight into everyday clinical practice. In fact, a separate real-world population-based study in New York state found that 2-year TURP and PUL retreatment occurred at 3.4% and 8.5%, respectively [19], corroborating the finding in this study that real-world retreatment occurred at higher rates than previously reported in RCTs. Prospective trials interrogate subjects for potential AEs, whereas a complication that did not result in outpatient treatment was not recorded. Clinical trials can also assess severity of AEs/complications through independent review of medical narrative, whereas that level of adjudication is not applicable to this database. Claims studies can also be vulnerable to losing patients to an insurer not captured within the database. However, comparisons to the more stable Medicare population largely corroborated overall results. It cannot be overlooked that this is a United States study focusing on procedures performed on an outpatient basis, and therefore these results may not necessarily reflect clinical practice in other regions where procedures are performed on an inpatient basis. Although medication data were provided within this database, a separate study is required to understand real-world BPH medication usage and its impact on postprocedural events.

Large-scale real-world analyses reflect event rates occurring in a diverse population of patients, providers, and healthcare settings in clinical settings. These results may provide insights into treatment invasiveness at the national level.

## Conclusions

This study showed that the 1-year surgical retreatment rate and adjusted hazard are similar among TURP, PVP, PUL, and WVTT. Five years post-index, retreatment rates were lowest for TURP and were similar between PVP and PUL. A key differentiator among BPH therapies is the 1-year risk of procedural complications, which were lowest after PUL and highest after WVTT. This large-scale analysis of real-world claims can assist patients and providers in better shared decision-making regarding BPH treatment.

### Supplementary information

Supplemental Material

## Data Availability

The data that support the findings are available from Merative but restrictions apply to the availability of these data, which were used under license for the current study, and so are not publicly available. Data are however available from the authors upon reasonable request and with permission of Merative.
