# Supplementary material for: Retreatment rates and postprocedural complications are higher than expected after BPH surgeries: a US healthcare claims and utilization study
Source: Prostate Cancer Prostatic Dis. 2023 Oct 26;27(3):485–91. doi: 10.1038/s41391-023-00741-8 (PMC11319193; doi:10.1038/s41391-023-00741-8)
Supplement: Supplementary file 1 — Supplemental Material [file 41391_2023_741_MOESM1_ESM.pdf]

Supplementary Tables

1. **Supplementary Table 1: Diagnostic codes**
2. **Supplementary Table 2: Index and surgical retreatment codes**
3. **Supplementary Table 3: Procedural complications codes**
4. **Supplementary Table 4: Comorbidities**
5. **Supplementary Table 5: CPT code frequencies**

Supplementary Table 1

ICD Code	Diagnosis	Code Description
R10	Abdominal and pelvic pain	abdominal and pelvic pain
R10.2	Abdominal and pelvic pain	pelvic and perineal pain
R10.30	Abdominal and pelvic pain	lower abdominal pain, unspecified
R10.31	Abdominal and pelvic pain	right lower quadrant pain
R10.32	Abdominal and pelvic pain	left lower quadrant pain
R10.33	Abdominal and pelvic pain	periumbilical pain
R10.8	Abdominal and pelvic pain	other abdominal pain
R10.9	Abdominal and pelvic pain	unspecified abdominal pain
789.0	Abdominal and pelvic pain	abdominal pain
R10.3	Abdominal and pelvic pain	pain localized to other parts of lower abdomen
789.07	Abdominal and pelvic pain	abdominal pain, generalized
G89.29	Abdominal and pelvic pain	other chronic pain
G89.4	Abdominal and pelvic pain	chronic pain syndrome
R52	Abdominal and pelvic pain	pain, unspecified
G89.28	Abdominal and pelvic pain	other chronic postprocedural pain
R39.82	Abdominal and pelvic pain	chronic bladder pain
G89.2	Abdominal and pelvic pain	chronic pain, not elsewhere classified
N45.4	Abscess of epididymis or testis	Abscess of epididymis or testis
N41.2	Abscess of prostate	Abscess of prostate
N99.4	Abscess of prostate	postprocedural pelvic peritoneal adhesions
601.2	Abscess of prostate	Abscess of prostate
N32.0	Bladder neck obstruction	bladder-neck obstruction
600.0	Benign Prostatic Hyperplasia	Hypertrophy (benign) of prostate without urinary obstruction and other lower urinary tract symptom (LUTS)
600.01	Benign Prostatic Hyperplasia	Hypertrophy (benign) of prostate without urinary obstruction and other lower urinary tract symptom (LUTS)
600.10	Benign Prostatic Hyperplasia	Nodular prostate
600.11	Benign Prostatic Hyperplasia	Nodular prostate with urinary obstruction
N40.0	Benign Prostatic Hyperplasia	Benign prostatic hyperplasia without lower urinary tract symptoms
N40.1	Benign Prostatic Hyperplasia	Benign prostatic hyperplasia with lower urinary tract symptoms
N40.2	Benign Prostatic Hyperplasia	Nodular prostate with lower urinary tract symptoms
N40.3	Benign Prostatic Hyperplasia	Nodular prostate without lower urinary tract symptoms
A4310	Catheterization	Insertion tray without drainage bag and without catheter
A4311	Catheterization	Insertion tray without drainage bag with indwelling catheter, Foley type, two-way latex with coating (Teflon, silicone, silicone elastomer or hydrophilic, etc.)
A4312	Catheterization	Insertion tray without drainage bag with indwelling catheter, Foley type, two-way, all silicone
A4313	Catheterization	Insertion tray without drainage bag with indwelling catheter, Foley type, three-way, for continuous irrigation

A4314	Catheterization	Insertion tray with drainage bag with indwelling catheter, Foley type, two-way latex with coating (Teflon, silicone, silicone elastomer or hydrophilic, etc.)
A4315	Catheterization	Insertion tray with drainage bag with indwelling catheter, Foley type, two-way, all silicone
A4316	Catheterization	Insertion tray with drainage bag with indwelling catheter, Foley type, three-way, for continuous irrigation
A4320	Catheterization	Irrigation tray with bulb or piston syringe, any purpose
A4321	Catheterization	Therapeutic agent for urinary catheter irrigation
A4322	Catheterization	Irrigation syringe, bulb or piston, each
A4326	Catheterization	Male external catheter with integral collection chamber, any type, each
T38.0	Catheter complications	Mechanical complication of urinary catheter
T83.011S	Catheter complications	Breakdown (mechanical) of indwelling urethral catheter, sequela
T83.098	Catheter complications	Other mechanical complication of other urinary catheter
T83.098S	Catheter complications	Other mechanical complication of other urinary catheter, sequela
T83.511A	Catheter complications	Infection and inflammatory reaction due to indwelling urethral catheter, initial encounter
T83511	Catheter complications	Infection and inflammatory reaction due to indwelling urethral catheter
T83511S	Catheter complications	Infection and inflammatory reaction due to indwelling urethral catheter, sequela
996.31	Catheter complications	Mechanical complication of genitourinary devices, implants and grafts due to indwelling catheter
996.64	Catheter complications	mechanical complication of internal orthopedic device, implant and graft
N30.00	Cystitis	Acute cystitis without hematuria
N30.80	Cystitis	Other cystitis without hematuria
N30.20	Cystitis	Other chronic cystitis without hematuria
N30.90	Cystitis	Cystitis, unspecified without hematuria
N30.01	Cystitis	Acute cystitis with hematuria
N30.81	Cystitis	Other cystitis with hematuria
N30.21	Cystitis	Other chronic cystitis with hematuria
N30.91	Cystitis	Cystitis, unspecified with hematuria
N30.10	Cystitis	Interstitial cystitis (chronic) without hematuria
N30.11	Cystitis	Interstitial cystitis (chronic) with hematuria
N30.30	Cystitis	Trigonitis without hematuria
N30.31	Cystitis	Trigonitis with hematuria
A06.81	Cystitis	amebic cystitis
A36.85	Cystitis	diphtheritic cystitis
A56.01	Cystitis	chlamydial cystitis and urethritis
A59.03	Cystitis	thrichomonal cystitis and urethritis
B3741	Cystitis	candidal cystitis and urethritis
B3749	Cystitis	other urogenital candidiasis
N309	Cystitis	cystitis, unspecified
595.0	Cystitis	Acute cystitis
595.81	Cystitis	Cystitis cystica
595.2	Cystitis	Other Chronic cystitis
595.9	Cystitis	Cystitis unspecified
596	Disorder urinary system_bladder issues	other disorders of bladder

596.51	Disorder urinary system_bladder issues	hypertonicity of bladder
N399	Disorder urinary system_bladder issues	Disorder of urinary system, unspecified
N32.8	Disorder urinary system_bladder issues	other specified disorders of bladder
N32.89	Disorder urinary system_bladder issues	other specified disorders of bladder
596.89	Disorder urinary system_bladder issues	other specified disorders of bladder
R30	Dysuria	Pain associated with micturition
R30.0	Dysuria	dysuria
R30.9	Dysuria	painful micturition, unspecified
788.1	Dysuria	dysuria
N53.11	Ejaculatory Dysfunction	Retarded ejaculation
N53.12	Ejaculatory Dysfunction	Painful ejaculation
N53.13	Ejaculatory Dysfunction	Anejaculatory orgasm
N53.14	Ejaculatory Dysfunction	Retrograde ejaculation
N53.19	Ejaculatory Dysfunction	Other ejaculatory dysfunction
608.87	Ejaculatory Dysfunction	Retrograde ejaculation
N45.1	Epididymitis	Epididymitis
A54.00	Epididymitis	gonococcal infection of lower genitourinary tract, unspecified
A59.00	Epididymitis	Urogenital trichomoniasis, unspecified
N45	Epididymo-Orchitis	orchitis and epididymitis
N45.3	Epididymo-Orchitis	Epididymo-orchitis
604.9	Epididymo-Orchitis	Other orchitis, epididymitis, and epididymo-orchitis, without mention of abscess
N52.1	Erectile Dysfunction	Erectile dysfunction due to diseases classified elsewhere
N52.2	Erectile Dysfunction	Drug-induced erectile dysfunction
N52.8	Erectile Dysfunction	Other male erectile dysfunction
N52.9	Erectile Dysfunction	Male erectile dysfunction, unspecified
N52.33	Erectile Dysfunction	Erectile dysfunction following urethral surgery
N52.37	Erectile Dysfunction	Erectile dysfunction following prostate ablative therapy
N52.39	Erectile Dysfunction	Other and unspecified postprocedural erectile dysfunction
R15.0	Fecal Incontinence	incomplete defecation
R15.1	Fecal Incontinence	fecal smearing
R15.2	Fecal Incontinence	fecal urgency
R15.9	Fecal Incontinence	full incontinence of feces
593.82	Fistula formation	Ureteral fistual
K60.0	Fistula formation	Acute anal fissure
K60.3	Fistula formation	Anal fistula
K60.4	Fistula formation	Rectal fistula
K60.5	Fistula formation	Anorectal fistula
N32.0	Fistula formation	Bladder-neck obstruction
N32.1	Fistula formation	Vesicointestinal fistula
N32.2	Fistula formation	Vesical fistula, not elsewhere classified
N36.0	Fistula formation	Urethral fistula

N36.1	Fistula formation	Urethral diverticulum
N36.2	Fistula formation	Urethral caruncle
565.0	Fistula formation	Anal fissure
565.1	Fistula formation	Anal fistula
596.0	Fistula formation	Bladder neck obstruction
596.1	Fistula formation	Intestinovesical fistula
596.2	Fistula formation	Vesical fistula NEC
599.1	Fistula formation	Urethral fistula
599.2	Fistula formation	Urethral diverticulum
599.3	Fistula formation	Urethral caruncle
A04.0	GI Infections	Enteropathogenic Escherichia coli infection
A04.8	GI Infections	Other specified bacterial intestinal infections
A09	GI Infections	Infectious gastroenteritis and colitis, unspecified
A04.1	GI Infections	Enterotoxigenic Escherichia coli infection
A04.2	GI Infections	Enteroinvasive Escherichia coli infection
A04.3	GI Infections	Enterohemorrhagic Escherichia coli infection
A04.4	GI Infections	Other intestinal Escherichia coli infections
A04.5	GI Infections	Campylobacter enteritis
A04.6	GI Infections	Enteritis due to Yersinia enterocolitica
A04.71	GI Infections	Enterocolitis due to Clostridium difficile, recurrent
A04.72	GI Infections	Enterocolitis due to Clostridium difficile, not specified as recurrent
008	GI Infections	Intestinal infections due to other organisms
008.00	GI Infections	Intestinal infections E. coli unspecified
008.01	GI Infections	Enteropathogenic Escherichia coli infection
008.1	GI Infections	Arizona enteritis
008.2	GI Infections	Aerobacter enteritis
008.3	GI Infections	Proteus enteritis
008.42	GI Infections	Pseudomonas enteritis
008.46	GI Infections	Intes infec oth anerobes
008.47	GI Infections	Int inf oth grm neg bctr
008.49	GI Infections	Bacterial enteritis NEC
009.0	GI Infections	Infectious colitis enteritis NOS
009.1	GI Infections	Colitis, enteritis and gastroenteritis of presumed infection
009.2	GI Infections	Infectious diarrhea NOS
009.3	GI Infections	Diarrhea of infect orig
008.02	GI Infections	enterotoxigenix e. coli
008.03	GI Infections	enteroinvasive e. coli
008.04	GI Infections	enterohemorrhagic e. coli
008.09	GI Infections	other intestinal e. coli infections
008.43	GI Infections	campylobacter
008.44	GI Infections	yersinia enterocolitica
008.45	GI Infections	clostridium difficile
009.0	GI Infections	Infectious colitis enteritis NOS

009.1	GI Infections	Colitis, enteritis and gastroenteritis of presumed infection
009.2	GI Infections	Infectious diarrhea NOS
009.3	GI Infections	Diarrhea of infect orig
596.7	Hematoma or hemorrhage	Hemorrhage into bladder wall
602.1	Hematoma or hemorrhage	Congestion and hemorrhage of prostate
M79.81	Hematoma or hemorrhage	Nontraumatic hematoma of soft tissue
M79.89	Hematoma or hemorrhage	Other specified soft tissue disorders
N99.6	Hematoma or hemorrhage	Intraoperative hemorrhage and hematoma of a genitourinary system organ or structure complicating a procedure
L76.02	Hematoma or hemorrhage	Intraoperative hemorrhage and hematoma of skin and subcutaneous tissue complicating other procedure
L76.32	Hematoma or hemorrhage	Postprocedural hematoma of skin and subcutaneous tissue following other procedure
L76.34	Hematoma or hemorrhage	Postprocedural seroma of skin and subcutaneous tissue following other procedure
N99.84	Hematoma or hemorrhage	postprocedural hematoma and seroma of a genitourinary system organ or structure following a procedure
N99.840	Hematoma or hemorrhage	postprocedural hematoma of a genitourinary system organ or structure following a genitourinary system procedure
N99.842	Hematoma or hemorrhage	postprocedural seroma of a genitourinary system organ or structure following a genitourinary system procedure
N9962	Hematoma or hemorrhage	intraoperative hemorrhage and hematoma of a genitourinary system organ or structure complicating other procedure
N99.71	Hematoma or hemorrhage	accidental puncture and laceration of a genitourinary system organ or structure during a genitourinary system procedure
N99.820	Hematoma or hemorrhage	postprocedural hemorrhage of a genitourinary system organ or structure following a procedure
N99.61	Hematoma or hemorrhage	intraoperative hemorrhage and hematoma of a genitourinary system organ or structure complicating a genitourinary system procedure
N42.1	Hematoma or hemorrhage	Congestion and hemorrhage of prostate
729.92	Hematoma or hemorrhage	Nontrauma hema soft tiss
729.99	Hematoma or hemorrhage	Soft tissue disorder NEC
998.11	Hematoma or hemorrhage	Hemorrhage complic proc
998.12	Hematoma or hemorrhage	Hematoma complic proc
R36.1	Hematospermia	hematospermia
R31	Hematuria	hematuria
R31.0	Hematuria	gross hematuria
R31.1	Hematuria	Benign essential microscopic hematuria
R31.2	Hematuria	Other microscopic hematuria
R31.21	Hematuria	asymptomatic microscopic hematuria
R31.29	Hematuria	Other microscopic hematuria
R31.9	Hematuria	hematuria, unspecified
599.71	Hematuria	gross hematuria
599.72	Hematuria	microscopic hematuria
N39.42	Incontinence	Incontinence without sensory awareness
N39.45	Incontinence	Continuous leakage
R39.81	Incontinence	functional urinary incontinence
R32	Incontinence	unspecified urinary incontinence
N36.41	Incontinence	hypermobility of urethra
N36.42	Incontinence	intrinsic sphincter deficiency
N3643	Incontinence	combined hypermobility of urethra and intrinsic sphincter deficiency

N39.4	Incontinence	other specified urinary incontinence
N39.49	Incontinence	other specified urinary incontinence
N39.490	Incontinence	overflow incontinence
N39.492	Incontinence	postural (urinary) incontinence
N39.498	Incontinence	other specified urinary incontinence
788.34	Incontinence	Incontnce wo sensr aware
788.37	Incontinence	Continuous leakage
N39.46	Incontinence_mixed	Mixed incontinence
788.33	Incontinence_mixed	Mixed incontinence
N39.3	Incontinence_stress	Stress incontinence (female) (male)
788.32	Incontinence_stress	Stress incontinence male
N39.41	Incontinence_urge	Urge incontinence
788.31	Incontinence_urge	Urge incontinence
T81.4	Infection	Infection following a procedure
595.3	Inflammation bladder neck	Trigonitis
N49	Inflammation male genital organs	Inflammation male genital organs
N49.0	Inflammation male genital organs	inflammatory disorders of seminal vesicle
N49.8	Inflammation male genital organs	Inflammatory disorders of other specified male genital organs and multiple sites
N49.9	Inflammation male genital organs	Inflammartory disorder of unspecified male genital organ
S37	Injury urinary organs	injury of urinary and pelvic organs
S37.10X	Injury urinary organs	Unspecified injury of ureter
S37.10X	Injury urinary organs	Unspecified injury of ureter, sequela
S37.30	Injury urinary organs	Unspecified injury of urethra
S37.30XA	Injury urinary organs	Unspecified injury of urethra, initial encounter
S37.30XS	Injury urinary organs	Unspecified injury of urethra, sequela
S37.20	Injury urinary organs	Unspecified injury of bladder
S37.20XS	Injury urinary organs	unspecified injury of bladder, sequela
S37.22XA	Injury urinary organs	contusion of bladder, initial encounter
S37.22XS	Injury urinary organs	contusion of bladder, sequela
R39.15	Irritative symptoms (LUTS)	urgency of urination
R39.191	Irritative symptoms (LUTS)	need to immediately re-void
R30.1	Irritative symptoms (LUTS)	Vesical tenesmus
R35.1	Irritative symptoms (LUTS)	nocturia
R35.0	Irritative symptoms (LUTS)	frequency of micturition
788.63	Irritative symptoms (LUTS)	urgency of urination
788.43	Irritative symptoms (LUTS)	nocturia
788.41	Irritative symptoms (LUTS)	urinary frequency
N32.81	OAB	overactive bladder
R39.11	Obstructive symptoms (LUTS)	Hesitancy of micturition
R39.12	Obstructive symptoms (LUTS)	poor urinary stream
R39.13	Obstructive symptoms (LUTS)	splitting of urinary stream
R39.14	Obstructive symptoms (LUTS)	Feeling of incomplete bladder emptying
R39.16	Obstructive symptoms (LUTS)	straining to void

788.61	Obstructive symptoms (LUTS)	splitting of urinary stream
788.62	Obstructive symptoms (LUTS)	slowing of urinary stream
788.64	Obstructive symptoms (LUTS)	urinary hesitancy
788.65	Obstructive symptoms (LUTS)	straining on urination
788.21	Obstructive symptoms (LUTS)	incomplete bladder emptying
N45.2	Orchitis	Orchitis
N50.89	Other disorder or dysfunction of male genitals	other specified disorders of the male genital organs
N50.8	Other disorder or dysfunction of male genitals	other specified disorders of the male genital organs
N53.8	Other disorder or dysfunction of male genitals	other male sexual dysfunction
N53.9	Other disorder or dysfunction of male genitals	unspecified male sexual dysfunction
608.9	Other disorder or dysfunction of male genitals	Unspecified disorder of male genital organs
N39.43	Other urinary symptoms	Post-void dribbling
788.99	Other urinary symptoms	Oth symptm urinary systm
R39.9	Other urinary symptoms	Unspecified symptoms and signs involving the genitourinary system
R39.198	Other urinary symptoms	Other difficulties with micturition
R39.1	Other urinary symptoms	Other difficulties with micturition
R39	Other urinary symptoms	Other and unspecified symptom and signs involving the genitourinary system
N99	Postop complication of the genitourinary system	intraoperative and postprocedural complications and disorders of genitourinary system
N99.89	Postop complication of the genitourinary system	Other postprocedural complications and disorders of genitourinary system
N99.81	Postop complication of the genitourinary system	Other intraoperative complications of genitourinary system
997.5	Postop complication of the genitourinary system	Surg compl-urinary tract
T85	Postop complication of the genitourinary system	Complications of other internal prosthetic devices, implants and grafts
T85.5	Postop complication of the genitourinary system	Mechanical complication of gastrointestinal prosthetic devices, implants and grafts
N41.0	Prostatitis	Acute prostatitis
N41.1	Prostatitis	Chronic prostatitis
N41.3	Prostatitis	Prostatocystitis
N41.4	Prostatitis	Granulomatous prostatitis
N41.8	Prostatitis	Other inflammatory diseases of prostate
N41.9	Prostatitis	Inflammatory disease of prostate, unspecified
A18.14	Prostatitis	tuberculosis of prostate
A54.22	Prostatitis	gonococcal prostatitis
A59.02	Prostatitis	trichomonal prostatitis
601	Prostatitis	Inflammatory diseases of prostate
601.1	Prostatitis	Chronic prostatitis
601.3	Prostatitis	Prostatocystitis
601.8	Prostatitis	Prostatic inflam dis NEC
601.9	Prostatitis	Prostatitis NOS
N10	Pyelonephritis	Acute pyelonephritis
N13.0	Pyelonephritis	Hydronephrosis with ureteropelvic junction obstruction
N28.0	Pyelonephritis	Ischemia and infarction of kidney

N13.30	Pyelonephritis	Unspecified hydronephrosis
N13.4	Pyelonephritis	Hydroureter
N13.39	Pyelonephritis	Other hydronephrosis
A02.25	Pyelonephritis	salmonella pyelonephritis
N11.1	Pyelonephritis	chronic obstructive pyelonephritis
N11.8	Pyelonephritis	other chronic tubulo-interstitial nephritis
N12	Pyelonephritis	tubulo-interstitital nephritis, not specified as acute or chronic
590.1	Pyelonephritis	Acute pyelonephritis
590.10	Pyelonephritis	Acute pyelonephritis without lesion of renal medullary necrosis NOS
593.81	Pyelonephritis	Renal vascular disorder
591	Pyelonephritis	Hydronephrosis
593.5	Pyelonephritis	Hydroureter
K62.5	Rectal hemorrhage	Hemorrhage of anus and rectum
Y65.2	Rectal hemorrhage	Failure in suture or ligature during surgical operation
Y65.4	Rectal hemorrhage	Failure to introduce or to remove other tube or instrument
567.23	Rectal hemorrhage	Spontan bact peritonitis
E876.2	Rectal hemorrhage	Failure in suture
E876.4	Rectal hemorrhage	Fail introd/remove tube
N19	Renal (compromise/failure/increase creatinine)	Unspecified kidney failure
N17.0	Renal (compromise/failure/increase creatinine)	Acute kidney failure with tubular necrosis
N17.1	Renal (compromise/failure/increase creatinine)	Acute kidney failure with acute cortical necrosis
N17.2	Renal (compromise/failure/increase creatinine)	Acute kidney failure with medullary necrosis
N17.8	Renal (compromise/failure/increase creatinine)	Other acute kidney failure
N17.9	Renal (compromise/failure/increase creatinine)	Acute kidney failure, unspecified
N99.0	Renal (compromise/failure/increase creatinine)	Postprocedural (acute) (chronic) kidney failure
N28.0	Renal (compromise/failure/increase creatinine)	Ischemia and infarction of kidney
N99.0	Renal (compromise/failure/increase creatinine)	Postprocedural (acute) (chronic) kidney failure
586	Renal (compromise/failure/increase creatinine)	Renal failure NOS
584.5	Renal (compromise/failure/increase creatinine)	Ac kidny fail, tubr necr
583.6	Renal (compromise/failure/increase creatinine)	Renal cort necrosis NOS
584.7	Renal (compromise/failure/increase creatinine)	Ac kidny fail, medu necr
584.8	Renal (compromise/failure/increase creatinine)	Acute kidney failure NEC
584.9	Renal (compromise/failure/increase creatinine)	Acute kidney failure NOS
593.81	Renal (compromise/failure/increase creatinine)	Renal vascular disorder
E87.0	Transfusion	Hyperosmolality and hypernatremia
E87.1	Transfusion	Hypo-osmolality and hyponatremia
R36.9	Urethral discharge	urethral discharge, unspecified
R36.0	Urethral discharge	urethral discharge without blood
N35.011	Urethral Stricture	Post-traumatic bulbous urethral stricture

N35.012	Urethral Stricture	Post-traumatic membranous urethral stricture
N35.013	Urethral Stricture	Post-traumatic anterior urethral stricture
N35.014	Urethral Stricture	Post-traumatic urethral stricture, male, unspecified
N35.016	Urethral Stricture	Post-traumatic urethral stricture, male, overlapping sites
N35.812	Urethral Stricture	Other urethral bulbous stricture, male
N35.813	Urethral Stricture	Other membranous urethral stricture, male
N35.814	Urethral Stricture	Other anterior urethral stricture, male
N35.816	Urethral Stricture	Other urethral stricture, male, overlapping sites
N35.819	Urethral Stricture	Other urethral stricture, male, unspecified site
N35.912	Urethral Stricture	Unspecified bulbous urethral stricture, male
N35.913	Urethral Stricture	Unspecified membranous urethral stricture, male
N35.914	Urethral Stricture	Unspecified anterior urethral stricture, male
N35.916	Urethral Stricture	Unspecified urethral stricture, male, overlapping sites
N13.1	Urethral Stricture	Hydronephrosis with ureteral stricture, not elsewhere classified
598.1	Urethral Stricture	Traum urethral stricture
598.2	Urethral Stricture	Postop urethral strictur
N35	Urethral Stricture	urethral stricture
N35.0	Urethral Stricture	post-traumamtic urethral stricture
N35.1	Urethral Stricture	postinfective urethral stricture
N35.112	Urethral Stricture	postinfective bulbous urethral stricture, male
N35.114	Urethral Stricture	postinfective anterior urethral stricture, male
N35.8	Urethral Stricture	other urethral stricture
N35.9	Urethral Stricture	urethral stricture, unspecified
N99.1	Urethral Stricture	postprocedural urethral stricture
N99.11	Urethral Stricture	postprocedural urethral stricture, male
N99.116	Urethral Stricture	postprocedural urethral stricture, male, overlapping sites
N99.111	Urethral Stricture	postprocedural bulbous urethral stricture, male
N99.112	Urethral Stricture	postprocedural membranous urethral stricture, male
N99.113	Urethral Stricture	postprocedural anterior bulbous urethral stricture, male
N99.114	Urethral Stricture	postprocedural urethral stricture, male, unspecified
N99.115	Urethral Stricture	postprocedural fossa navicularis urethral stricture
N35.010	Urethral Stricture (meatal)	Post-traumatic urethral stricture, male, meatal
N35.811	Urethral Stricture (meatal)	Other urethral stricture, male, meatal
N35.911	Urethral Stricture (meatal)	Unspecified urethral stricture, male, meatal
N35.01	Urethral Stricture (meatal)	post-traumatic urethral stricture, male, meatal
N35.111	Urethral Stricture (meatal)	postinfective urethral stricture, male, meatal
N99.110	Urethral Stricture (meatal)	postprocedural urethral stricture, male, meatal
N20.1	Urinary Calculus (encrustation, stone, nephrolithiasis)	Calculus of ureter
N20.0	Urinary Calculus (encrustation, stone, nephrolithiasis)	Calculus of kidney
N21	Urinary Calculus (encrustation, stone, nephrolithiasis)	Calculus of lower urinary tract
N21.0	Urinary Calculus (encrustation, stone, nephrolithiasis)	Calculus in bladder

N21.1	Urinary Calculus (encrustation, stone, nephrolithiasis)	Calculus in urethra
N21.9	Urinary Calculus (encrustation, stone, nephrolithiasis)	Calculus of lower urinary tract, unspecified
N21.8	Urinary Calculus (encrustation, stone, nephrolithiasis)	Other lower urinary tract calculus
N42.0	Urinary Calculus (encrustation, stone, nephrolithiasis)	Calculus of prostate
N13.2	Urinary Calculus (encrustation, stone, nephrolithiasis)	Hydronephrosis with renal and ureteral calculous obstruction
T19	Urinary Calculus (encrustation, stone, nephrolithiasis)	foreign body in genitourinary tract
Z18.83	Urinary Calculus (encrustation, stone, nephrolithiasis)	retained stone or crystalline fragments
592	Urinary Calculus (encrustation, stone, nephrolithiasis)	Calculus of kidney and ureter
592.0	Urinary Calculus (encrustation, stone, nephrolithiasis)	Calculus of kidney
592.1	Urinary Calculus (encrustation, stone, nephrolithiasis)	Calculus of ureter
592.9	Urinary Calculus (encrustation, stone, nephrolithiasis)	Urinary calculus unspecified
274.11	Urinary Calculus (encrustation, stone, nephrolithiasis)	Uric acid nephrolithias
594	Urinary Calculus (encrustation, stone, nephrolithiasis)	Calculus of lower urinary tract
594.1	Urinary Calculus (encrustation, stone, nephrolithiasis)	Bladder calculus NEC
594.2	Urinary Calculus (encrustation, stone, nephrolithiasis)	Urethral calculus
594.8	Urinary Calculus (encrustation, stone, nephrolithiasis)	Lower urin calcul NEC
602.0	Urinary Calculus (encrustation, stone, nephrolithiasis)	Calculus of prostate
R33.8	Urinary Retention	Other retention of urine
R33.9	Urinary Retention	Retention of urine, unspecified
788.20	Urinary Retention	Retention of urine, unspecified
788.29	Urinary Retention	Other retention of urine
A26.7	Urosepsis	Erysipelothrix sepsis
A32.7	Urosepsis	Listerial sepsis
A40.0	Urosepsis	Sepsis due to streptococcus, group A
A40.1	Urosepsis	sepsis due to streptococcus, group B
A40.3	Urosepsis	sepsis due to streptococcus pneumoniae
A40.8	Urosepsis	other streptococcal sepsis
A40.9	Urosepsis	streptococcal sepsis, unspecified
A41.3	Urosepsis	sepsis due to hemophilus influenzae
A41.4	Urosepsis	sepsis due to anaerobes
A41.50	Urosepsis	gram-negative sepsis, unspecified
A41.51	Urosepsis	sepsis due to e.coli
A41.52	Urosepsis	sepsis due to pseudomonas
A41.53	Urosepsis	sepsis due to serratia
A41.59	Urosepsis	other gram-negative sepsis
A42.7	Urosepsis	actinomycotic sepsis
B37.7	Urosepsis	candidal sepsis
O85	Urosepsis	leishmaniasis

R65.21	Urosepsis	severe sepsis with septic shock
R78.81	Urosepsis	bacteremia
R65.2	Urosepsis	severe sepsis
A41.9	Urospesis	Sepsis, unspecified organism
A54.8	Urospesis	Other gonococcal infections
A41.8	Urospesis	Other specified sepsis
038.9	Urospesis	Unspecified septicemia
A02.1	Urospesis	Salmonella sepsis
599.0	UTI	Urinary tract infection, site not specified
599.89	UTI	Other specified disorders of urinary tract
N39.0	UTI	Urinary tract infection, site not specified
N39.8	UTI	Other specified disorders of urinary system

Supplementary Table 2

CPT / ICD 10 PCS code	Procedure	Code description
52648	GreenLight	Laser vaporization of prostate, including control of postoperative bleeding, complete (vasectomy, meatotomy, cystourethroscopy, urethral calibration and/or dilation, internal urethrotomy and transurethral resection of prostate are included if performed)
DVY0KZZ	GreenLight	Laser Interstitial Thermal Therapy of Prostate
52649	HoLEP	Laser enucleation of the prostate with morcellation, including control of postoperative bleeding, complete (vasectomy, meatotomy, cystourethroscopy, urethral calibration and/or dilation, internal urethrotomy and transurethral resection of prostate are included if performed)
55720	Infection	Prostatotomy, external drainage of prostatic abscess, any approach; simple
52441	PUL	Cystourethroscopy, with insertion of permanent adjustable transprostatic implant; single implant
52442	PUL	Cystourethroscopy, with insertion of permanent adjustable transprostatic implant; each additional permanent adjustable transprostatic implant (List separately in addition to code for primary procedure)
C9739	PUL	Cystourethroscopy, with insertion of transprostatic implant; one to three implants
C9740	PUL	Cystourethroscopy, with insertion of transprostatic implant; four or more implants
53899	Rezum	Unlisted procedure, urinary system
53854	Rezum	Transurethral destruction of prostate tissue; by radiofrequency water vapor (steam) thermal therapy
C9748	Rezum	Transurethral destruction of prostate tissue; by radiofrequency water vapor (steam) thermal therapy
52450	TUIP/Bladder neck repair	Transurethral incision of the prostate
55705	TUIP/Bladder neck repair	Biopsy, prostate; incisional, any approach
52500	TURP	Transurethral resection of bladder neck (separate procedure)
52640	TURP	Transurethral resection; of postoperative bladder neck contracture
0VT08ZZ	TURP	Resection of Prostate, Via Natural or Artificial Opening Endoscopic
52601	TURP	Transurethral electrosurgical resection of prostate, including control of postoperative bleeding, complete (vasectomy, meatotomy, cystourethroscopy, urethral calibration and/or dilation, and internal urethrotomy are included)
52614	TURP	Transurethral resection of prostate; second stage of two-stage resection (resection completed) DELETED CODE
52620	TURP	Transurethral resection; of residual obstructive tissue after 90 days postoperative DELETED CODE
52630	TURP	Transurethral resection; residual or regrowth of obstructive prostate tissue including control of postoperative bleeding, complete (vasectomy, meatotomy, cystourethroscopy, urethral calibration and/or dilation, and internal urethrotomy are included)

Supplementary Table 3

CPT / ICD 10 PCS	Procedure	Code description
51700	Bladder Irrigation	Bladder irrigation, simple, lavage and/or instillation
51520	Bladder Neck Repair	Cystotomy; for simple excision of vesical neck (separate procedure)
51800	Bladder Neck Repair	Cystoplasty or cystourethroplasty, plastic operation on bladder and/or vesical neck (anterior Y-plasty, vesical fundus resection), any procedure, with or without wedge resection of posterior vesical neck
0TQC0ZZ	Bladder neck repair	Bladder neck repair
0TQC3ZZ	Bladder neck repair	Bladder neck repair
0TQC4ZZ	Bladder neck repair	Bladder neck repair
0TQC7ZZ	Bladder neck repair	Bladder neck repair
0TQC8ZZ	Bladder neck repair	Bladder neck repair
52647	Bleeding Control/LCP	Laser coagulation of prostate, including control of postoperative bleeding, complete (vasectomy, meatotomy, cystourethroscopy, urethral calibration and/or dilation, and internal urethrotomy are included if performed)
51701	Catheterization	Insertion of non-indwelling bladder catheter (eg, straight catheterization for residual urine)
51702	Catheterization	Insertion of temporary indwelling bladder catheter; simple (eg, Foley)
51703	Catheterization	Insertion of temporary indwelling bladder catheter; complicated (eg, altered anatomy, fractured catheter/balloon)
51102	Catheterization	Aspiration of bladder; with insertion of suprapubic catheter
52001-59	Clot Removal	Cystourethroscopy with irrigation and evacuation of multiple obstructing clots
52351	Cystoscopy	Cystourethroscopy, with ureteroscopy and/or pyeloscopy; diagnostic
52000	Cystoscopy	Cystourethroscopy (separate procedure)
52283	Cystoscopy and Stricture Repair	Cystourethroscopy, with steroid injection into stricture
52276	Cystoscopy and Stricture Repair	Cystourethroscopy with direct vision internal urethrotomy
52281	Cystoscopy and Stricture Repair	Cystourethroscopy, with calibration and/or dilation of urethral stricture or stenosis, with or without meatotomy, with or without injection procedure for cystography, male or female
53600	Cystoscopy and Stricture Repair	Dilation of urethral stricture by passage of sound or urethral dilator, male
53601	Cystoscopy and Stricture Repair	Dilation of urethral stricture by passage of sound or urethral dilator, male; subsequent
53605	Cystoscopy and Stricture Repair	Dilation of urethral stricture or vesical neck by passage of sound or urethral dilator, male, general or conduction (spinal) anesthesia
53620	Cystoscopy and Stricture Repair	Dilation of urethral stricture by passage of filiform and follower, male; initial
53621	Cystoscopy and Stricture Repair	Dilation of urethral stricture by passage of filiform and follower, male; subsequent
52341	Cystoscopy and Stricture Repair	Cystourethroscopy; with treatment of ureteral stricture (eg, balloon dilation, laser, electrocautery, and incision)
52342	Cystoscopy and Stricture Repair	Cystourethroscopy; with treatment of ureteropelvic junction stricture (eg, balloon dilation, laser, electrocautery, and incision)
52344	Cystoscopy and Stricture Repair	Cystourethroscopy with ureteroscopy; with treatment of ureteral stricture (eg, balloon dilation, laser, electrocautery, and incision)

52345	Cystoscopy and Stricture Repair	Cystourethroscopy with ureteroscopy; with treatment of ureteropelvic junction stricture (eg, balloon dilation, laser, electrocautery, and incision)
52346	Cystoscopy and Stricture Repair	Cystourethroscopy with ureteroscopy; with treatment of intra-renal stricture (eg, balloon dilation, laser, electrocautery, and incision)
52260	Cystoscopy for Bladder Dilation	Cystourethroscopy, with dilation of bladder for interstitial cystitis; general or conduction (spinal) anesthesia
52265	Cystoscopy for Bladder Dilation	Cystourethroscopy, with dilation of bladder for interstitial cystitis; local anesthesia
52204	Cystoscopy with Biopsy	Cystourethroscopy, with biopsy(s)
52005	Cystoscopy with Catheterization	Cystourethroscopy, with ureteral catheterization, with or without irrigation, instillation, or ureteropyelography, exclusive of radiologic service;
52007	Cystoscopy with Catheterization	Cystourethroscopy, with ureteral catheterization, with or without irrigation, instillation, or ureteropyelography, exclusive of radiologic service; with brush biopsy of ureter and/or renal pelvis
52327	Cystoscopy with Catheterization	Cystourethroscopy (including ureteral catheterization); with subureteric injection of implant material
52224	Cystoscopy with Fulguration	Cystourethroscopy, with fulguration (including cryosurgery or laser surgery) or treatment of MINOR (less than 0.5 cm) lesion(s) with or without biopsy
52250	Cystoscopy with Fulguration	Cystourethroscopy with insertion of radioactive substance, with or without biopsy or fulguration
52275	Cystoscopy with Urethral Incision	Cystourethroscopy, with internal urethrotomy; male
52277	Cystoscopy with Urethral Incision	Cystourethroscopy, with resection of external sphincter (sphincterotomy)
52214	Fulguration	Cystourethroscopy, with fulguration (including cryosurgery or laser surgery) of trigone, bladder neck, prostatic fossa, urethra, or periurethral glands
0T5D0ZZ	Fulguration	Destruction of Urethra
0T5D3ZZ	Fulguration	Destruction of Urethra
0T5D4ZZ	Fulguration	Destruction of Urethra
0T5D7ZZ	Fulguration	Destruction of Urethra
0T5D8ZZ	Fulguration	Destruction of Urethra
0T5DXZZ	Fulguration	Destruction of Urethra
53440	Incontinence	Sling operation for correction of male urinary incontinence
53442	Incontinence	Removal or revision of sling for male urinary incontinence
53445	Incontinence	Insertion of inflatable urethral/bladder neck sphincter, including placement of pump, reservoir, and cuff
53446	Incontinence	Removal of inflatable urethral/bladder neck sphincter, including pump, reservoir, and cuff
53449	Incontinence	Repair of inflatable urethral/bladder neck sphincter, including pump, reservoir, and cuff
53447	Incontinence	Removal and replacement of inflatable urethral/bladder neck sphincter including pump, reservoir, and cuff at the same operative session
53448	Incontinence	Removal and replacement of inflatable urethral/bladder neck sphincter including pump, reservoir, and cuff through an infected field at the same operative session including irrigation and debridement of infected tissue

51715	Incontinence	Endoscopic injection of implant material into the submucosal tissues of the urethra and/or bladder neck
0TUC8JZ	Incontinence	Supplement bladder neck with synthetic substitute, via natural or artificial opening endoscopic
0TUD8JZ	Incontinence	Supplement urethra with synthetic substitute, via natural or artificial opening endoscopic
3E0K3GC	Incontinence	Introduction of other therapeutic substance into genitourinary tract, percutaneous approach
3E0K8GC	Incontinence	Introduction of other therapeutic substance into genitourinary tract, via natural or artificial opening endoscopic
0THC0LZ	Incontinence_Artificial sphincter	Insertion of artificial sphincter into bladder neck
0THC3LZ	Incontinence_Artificial sphincter	Insertion of artificial sphincter into bladder neck
0THC4LZ	Incontinence_Artificial sphincter	Insertion of artificial sphincter into bladder neck
0THC7LZ	Incontinence_Artificial sphincter	Insertion of artificial sphincter into bladder neck
0THC8LZ	Incontinence_Artificial sphincter	Insertion of artificial sphincter into bladder neck
0THD0LZ	Incontinence_Artificial sphincter	Insertion of artificial sphincter into bladder neck
0THD3LZ	Incontinence_Artificial sphincter	Insertion of artificial sphincter into bladder neck
0THD4LZ	Incontinence_Artificial sphincter	Insertion of artificial sphincter into bladder neck
0THD7LZ	Incontinence_Artificial sphincter	Insertion of artificial sphincter into bladder neck
0THD8LZ	Incontinence_Artificial sphincter	Insertion of artificial sphincter into bladder neck
0THDXLZ	Incontinence_Artificial sphincter	Insertion of artificial sphincter into bladder neck
55720	Infection	Prostatotomy, external drainage of prostatic abscess, any approach; simple
55725	Infection	Prostatotomy, external drainage of prostatic abscess, any approach.
0V9000Z	Infection_drain	Drainage of Prostate
0V900ZX	Infection_drain	Drainage of Prostate
0V900ZZ	Infection_drain	Drainage of Prostate
0V9030Z	Infection_drain	Drainage of Prostate
0V903ZX	Infection_drain	Drainage of Prostate
0V903ZZ	Infection_drain	Drainage of Prostate
0V9040Z	Infection_drain	Drainage of Prostate
0V904ZX	Infection_drain	Drainage of Prostate
0V904ZZ	Infection_drain	Drainage of Prostate
0V9070Z	Infection_drain	Drainage of Prostate
0V907ZX	Infection_drain	Drainage of Prostate
0V907ZZ	Infection_drain	Drainage of Prostate
0V9080Z	Infection_drain	Drainage of Prostate
0V908ZX	Infection_drain	Drainage of Prostate
0V908ZZ	Infection_drain	Drainage of Prostate

50432	Nephrostomy	Placement of nephrostomy catheter, percutaneous, including diagnostic nephrostogram and/or ureterogram when performed, imaging guidance (eg, ultrasound and/or fluoroscopy) and all associated radiologic supervision and interpretation.
52334	Nephrostomy	Cystourethroscopy with insertion of ureteral guide wire through kidney to establish a percutaneous nephrostomy, retrograde
51050	Stone Removal	Cystolithotomy, cystotomy with removal of calculus, without vesical neck resection
52310	Stone Removal	Cystourethroscopy, with removal of foreign body, calculus, or ureteral stent from urethra or bladder (separate procedure); simple
52315	Stone Removal	Cystourethroscopy, with removal of foreign body, calculus, or ureteral stent from urethra or bladder (separate procedure); complicated
52317	Stone Removal	Litholapaxy: crushing or fragmentation of calculus by any means in bladder and removal of fragments; simple or small (less than 2.5 cm)
52318	Stone Removal	Litholapaxy: crushing or fragmentation of calculus by any means in bladder and removal of fragments; complicated or large (over 2.5 cm)
0T7C0DZ	Stricture_Bladder Neck Contracture	Dilation Bladder Neck
0T7C0ZZ	Stricture_Bladder Neck Contracture	Dilation Bladder Neck
0T7C3DZ	Stricture_Bladder Neck Contracture	Dilation Bladder Neck
0T7C3ZZ	Stricture_Bladder Neck Contracture	Dilation Bladder Neck
0T7C4DZ	Stricture_Bladder Neck Contracture	Dilation Bladder Neck
0T7C4ZZ	Stricture_Bladder Neck Contracture	Dilation Bladder Neck
0T7C7DZ	Stricture_Bladder Neck Contracture	Dilation Bladder Neck
0T7C7ZZ	Stricture_Bladder Neck Contracture	Dilation Bladder Neck
0T7C8DZ	Stricture_Bladder Neck Contracture	Dilation Bladder Neck
0T7C8ZZ	Stricture_Bladder Neck Contracture	Dilation Bladder Neck
36430	Transfusion	Transfusion, blood or blood components
52450	TUIP/Bladder neck repair	Transurethral incision of the prostate
55705	TUIP/Bladder neck repair	Biopsy, prostate; incisional, any approach
50947	Ureteral Reimplantation	Laparoscopy, surgical; ureteroneocystostomy with cystoscopy and ureteral stent placement
50948	Ureteral Reimplantation	Laparoscopy, surgical; ureteroneocystostomy without cystoscopy and ureteral stent placemen
0TQD0ZZ	Urethral Repair	Repair Urethra
0TQD3ZZ	Urethral Repair	Repair Urethra
0TQD4ZZ	Urethral Repair	Repair Urethra
0TQD7ZZ	Urethral Repair	Repair Urethra
0TQD8ZZ	Urethral Repair	Repair Urethra
0TQDXZZ	Urethral Repair	Repair Urethra
53415	Urethral Repairs	Urethroplasty, transpubic or perineal, 1-stage, for reconstruction or repair of prostatic or membranous urethra
53420	Urethral Repairs	Urethroplasty, 2-stage reconstruction or repair of prostatic or membranous urethra; first stage
53425	Urethral Repairs	Urethroplasty, 2-stage reconstruction or repair of prostatic or membranous urethra; second stage

53020	Urethral Repairs	Meatotomy, cutting of meatus (separate procedure)
53510	Urethral Repairs	Urethrorrhaphy, suture of urethral wound or injury; perineal
53515	Urethral Repairs	Urethrorrhaphy, suture of urethral wound or injury; prostatomembranous
53855	Urethral Stent Placement	Insertion of a temporary prostatic urethral stent, including urethral measurement
52282	Urethral Stent Placement	Cystourethroscopy, with insertion of permanent urethral stent
0T7D0DZ	Urethral Stricture	Dilaton of Urethra
0T7D0ZZ	Urethral Stricture	Dilaton of Urethra
0T7D3DZ	Urethral Stricture	Dilaton of Urethra
0T7D3ZZ	Urethral Stricture	Dilaton of Urethra
0T7D4DZ	Urethral Stricture	Dilaton of Urethra
0T7D4ZZ	Urethral Stricture	Dilaton of Urethra
0T7D7DZ	Urethral Stricture	Dilaton of Urethra
0T7D7ZZ	Urethral Stricture	Dilaton of Urethra
0T7D8DZ	Urethral Stricture	Dilaton of Urethra
0T7D8ZZ	Urethral Stricture	Dilaton of Urethra

Supplementary Table 4

	TURP	GreenLight PVP	UroLift PUL	Rezum WVTT	p-value
Abdominal and pelvic pain	81/22631 (.36%)	35/11391(.31%)	29/7531 (.39%)	2/1597 (.13%)	0.4
Abscess of epididymis or testis	0/0(0%)	0/0(0%)	0/0(0%)	0/0(0%)	1.0
Abscess of prostate	33/22631 (.15%)	3/11391(.03%)	0/0(0%)	0/0(0%)	<.0001
Bladder neck obstruction	1080/22631 (4.8%)	499/11391(4.4%)	237/7531 (3.1%)	13/1597 (.81%)	<.0001
Catheter complications	0/0(0%)	0/0(0%)	0/0(0%)	0/0(0%)	<.0001
Cystitis	177/22631 (.78%)	73/11391(.64%)	25/7531 (.33%)	4/1597 (.25%)	<.0001
Disorder urinary system_bladder issues	826/22631 (3.6%)	407/11391(3.6%)	156/7531 (2.1%)	15/1597 (.94%)	<.0001
Dysuria	110/22631 (.49%)	54/11391(.47%)	37/7531 (.49%)	9/1597 (.56%)	0.9
Ejaculatory Dysfunction	7/22631 (.03%)	2/11391(.02%)	7/7531 (.09%)	2/1597 (.13%)	0.02
Epididymitis	5/22631 (.02%)	7/11391(.06%)	4/7531 (.05%)	1/1597 (.06%)	0.3
Epididymo-Orchitis	3/22631 (.01%)	2/11391(.02%)	1/7531 (.01%)	15/1597 (.94%)	<.0001
Erectile Dysfunction	154/22631 (.68%)	67/11391(.59%)	99/7531 (1.3%)	34/1597 (2.1%)	<.0001
Fecal Incontinence	0/0(0%)	0/0(0%)	0/0(0%)	0/0(0%)	<.0001
Fistula formation	9/22631 (.04%)	1/11391(.01%)	2/7531 (.03%)	2/1597 (.13%)	0.08
GI Infections	0/0(0%)	0/0(0%)	0/0(0%)	0/0(0%)	0.08
Hematoma or hemorrhage	36/22631 (.16%)	7/11391(.06%)	3/7531 (.04%)	0/0(0%)	0.005
Hematospermia	2/22631 (.01%)	3/11391(.03%)	4/7531 (.05%)	2/1597 (.13%)	0.01
Hematuria	0/0(0%)	0/0(0%)	0/0(0%)	0/0(0%)	1
Incontinence	95/22631 (.42%)	51/11391(.45%)	28/7531 (.37%)	6/1597 (.38%)	0.9
Incontinence mixed	0/0(0%)	0/0(0%)	0/0(0%)	0/0(0%)	1
Incontinence stress	0/0(0%)	0/0(0%)	0/0(0%)	0/0(0%)	1
Urge Incontinence	172/22631 (.76%)	109/11391(.96%)	94/7531 (1.2%)	15/1597 (.94%)	0.001
Infection	0/0(0%)	0/0(0%)	0/0(0%)	0/0(0%)	1.0
Inflammation bladder neck	0/0(0%)	1/11391(.01%)	0/0(0%)	0/0(0%)	0.4
Inflammation male genital organs	1/22631 (.00%)	0/0(0%)	1/7531 (.01%)	2/1597 (.13%)	<.0001
Injury urinary organs	2/22631 (.01%)	0/0(0%)	0/0(0%)	0/0(0%)	0.6

Irritative symptoms (LUTS)	1919/22631 (8.5%)	1287/11391(11%)	1504/7531 (20%)	274/1597 (17%)	<.0001
OAB	122/22631 (.54%)	61/11391(.54%)	45/7531 (.60%)	5/1597 (.31%)	0.6
Obstructive symptoms (LUTS)	1711/22631 (7.6%)	1145/11391(10%)	1090/7531 (14%)	183/1597 (11%)	<.0001
Orchitis	3/22631 (.01%)	1/11391(.01%)	1/7531 (.01%)	0/0(0%)	0.9
Other disorder or dysfunction of male genitals	8/22631 (.04%)	3/11391(.03%)	2/7531 (.03%)	0/0(0%)	0.9
Other urinary symptoms	225/22631 (.99%)	123/11391(1.1%)	154/7531 (2.0%)	27/1597 (1.7%)	<.0001
Postop complication of the genitourinary system	8/22631 (.04%)	3/11391(.03%)	1/7531 (.01%)	1/1597 (.06%)	0.7
Prostatitis	1098/22631 (4.9%)	92/11391(.81%)	40/7531 (.53%)	7/1597 (.44%)	<.0001
Pyelonephritis	292/22631 (1.3%)	92/11391(.81%)	15/7531 (.20%)	2/1597 (.13%)	<.0001
Rectal hemorrhage	1/22631 (.00%)	2/11391(.02%)	0/0(0%)	0/0(0%)	0.4
Renal (compromise/failure/increase creatinine)	57/22631 (.25%)	26/11391(.23%)	2/7531 (.03%)	1/1597 (.06%)	0.0009
Transfusion	15/22631 (.07%)	2/11391(.02%)	1/7531 (.01%)	0/0(0%)	0.07
Urethral discharge	0/0(0%)	0/0(0%)	0/0(0%)	0/0(0%)	1
Urethral Stricture	438/22631 (1.9%)	210/11391(1.8%)	64/7531 (.85%)	8/1597 (.50%)	<.0001
Urethral Stricture (meatal)	69/22631 (.30%)	25/11391(.22%)	11/7531 (.15%)	2/1597 (.13%)	0.06
Urinary Calculus (encrustation, stone, nephrolithiasis)	1538/22631 (6.8%)	672/11391(5.9%)	120/7531 (1.6%)	25/1597 (1.6%)	<.0001
Urinary Retention	5205/22631 (23%)	2372/11391(21%)	795/7531 (11%)	152/1597 (9.5%)	<.0001
Urospesis	5/22631 (.02%)	0/0(0%)	0/0(0%)	0/0(0%)	0.2
UTI	279/22631 (1.2%)	113/11391(.99%)	50/7531 (.66%)	13/1597 (.81%)	0.0002
Coagulopathy	0/0(0%)	0/0(0%)	0/0(0%)	0/0(0%)	1.0
Diabetes	1911/22631 (8.4%)	871/11391(7.6%)	417/7531 (5.5%)	18/1597 (1.1%)	<.0001
Hematuria	896/22631 (4.0%)	295/11391(2.6%)	90/7531 (1.2%)	55/1597 (3.4%)	<.0001
Kidney Disease	233/22631 (1.0%)	114/11391(1.0%)	38/7531 (.50%)	3/1597 (.19%)	<.0001
Parkinson Disease	84/22631 (.37%)	28/11391(.25%)	16/7531 (.21%)	1/1597 (.06%)	0.02
Prostate Cancer	584/22631 (2.6%)	124/11391(1.1%)	48/7531 (.64%)	18/1597 (1.1%)	<.0001

Supplementary Table 5:

	TURP	GreenLight PVP	UroLift PUL	Rezum WVTT	Log Rank p-value
Proportion of overall procedural complications	17%<br>(3545, 100.0%)	19%<br>(2066, 100.0%)	15%<br>(1119, 100.0%)	26%<br>(335, 100.0%)	<.0001
Bladder Irrigation	7.11%<br>(1546, 100.0%)	6.96%<br>(768, 100.0%)	6.62%<br>(496, 100.0%)	11.97%<br>(159, 100.0%)	<.0001
51700	7.11%<br>(1546, 100.0%)	6.96%<br>(768, 100.0%)	6.62%<br>(496, 100.0%)	11.97%<br>(159, 100.0%)	<.0001
Bladder Neck Repairs	0.03%<br>(7, 100.0%)	0.05%<br>(6, 100.0%)	0.11%<br>(8, 100.0%)	0.0%<br>(0,0.0%)	0.0769
51520	0.01%<br>(2, 28.57%)	0.00%<br>(0, 0.00%)	0.00%<br>(0, 0.00%)	0.0%<br>(0,0.0%)	0.6092
51800	0.00%<br>(0, 0.00%)	0.00%<br>(0, 0.00%)	0.00%<br>(0, 0.00%)	0.0%<br>(0,0.0%)	1.0000
0TQC0ZZ	0.00%<br>(0, 0.00%)	0.00%<br>(0, 0.00%)	0.00%<br>(0, 0.00%)	0.0%<br>(0,0.0%)	1.0000
0TQC3ZZ	0.00%<br>(0, 0.00%)	0.00%<br>(0, 0.00%)	0.00%<br>(0, 0.00%)	0.0%<br>(0,0.0%)	1.0000
0TQC4ZZ	0.00%<br>(0, 0.00%)	0.00%<br>(0, 0.00%)	0.00%<br>(0, 0.00%)	0.0%<br>(0,0.0%)	1.0000
0TQC7ZZ	0.00%<br>(0, 0.00%)	0.00%<br>(0, 0.00%)	0.00%<br>(0, 0.00%)	0.0%<br>(0,0.0%)	1.0000
0TQC8ZZ	0.00%<br>(0, 0.00%)	0.00%<br>(0, 0.00%)	0.00%<br>(0, 0.00%)	0.0%<br>(0,0.0%)	1.0000
52260	0.02%<br>(4, 57.14%)	0.03%<br>(3, 50.00%)	0.09%<br>(7, 87.50%)	0.0%<br>(0,0.0%)	0.0182
52265	0.00%<br>(0, 0.00%)	0.00%<br>(0, 0.00%)	0.01%<br>(1, 12.50%)	0.0%<br>(0,0.0%)	0.2078
52450	0.00%<br>(0, 0.00%)	0.00%<br>(0, 0.00%)	0.00%<br>(0, 0.00%)	0.0%<br>(0,0.0%)	1.0000
55705	0.01%<br>(2, 28.57%)	0.03%<br>(3, 50.00%)	0.00%<br>(0, 0.00%)	0.0%<br>(0,0.0%)	0.3452
0T7C0DZ	0.00%<br>(0, 0.00%)	0.00%<br>(0, 0.00%)	0.00%<br>(0, 0.00%)	0.0%<br>(0,0.0%)	1.0000
0T7C0ZZ	0.00%<br>(0, 0.00%)	0.00%<br>(0, 0.00%)	0.00%<br>(0, 0.00%)	0.0%<br>(0,0.0%)	1.0000
0T7C3DZ	0.00%<br>(0, 0.00%)	0.00%<br>(0, 0.00%)	0.00%<br>(0, 0.00%)	0.0%<br>(0,0.0%)	1.0000
0T7C3ZZ	0.00%<br>(0, 0.00%)	0.00%<br>(0, 0.00%)	0.00%<br>(0, 0.00%)	0.0%<br>(0,0.0%)	1.0000
0T7C4DZ	0.00%<br>(0, 0.00%)	0.00%<br>(0, 0.00%)	0.00%<br>(0, 0.00%)	0.0%<br>(0,0.0%)	1.0000
0T7C4ZZ	0.00%<br>(0, 0.00%)	0.00%<br>(0, 0.00%)	0.00%<br>(0, 0.00%)	0.0%<br>(0,0.0%)	1.0000
0T7C7DZ	0.00%<br>(0, 0.00%)	0.00%<br>(0, 0.00%)	0.00%<br>(0, 0.00%)	0.0%<br>(0,0.0%)	1.0000
0T7C7ZZ	0.00%<br>(0, 0.00%)	0.00%<br>(0, 0.00%)	0.00%<br>(0, 0.00%)	0.0%<br>(0,0.0%)	1.0000
0T7C8DZ	0.00%<br>(0, 0.00%)	0.00%<br>(0, 0.00%)	0.00%<br>(0, 0.00%)	0.0%<br>(0,0.0%)	1.0000
0T7C8ZZ	0.00%<br>(0, 0.00%)	0.00%<br>(0, 0.00%)	0.00%<br>(0, 0.00%)	0.0%<br>(0,0.0%)	1.0000
Bleeding Control	0.02%<br>(5, 100.0%)	0.93%<br>(103, 100.0%)	0.03%<br>(2, 100.0%)	0.0%<br>(0,0.0%)	<.0001

	TURP	GreenLight PVP	UroLift PUL	Rezum WVTT	Log Rank p-value
52647	0.02%<br>(5, 100.0%)	0.93%<br>(103, 100.0%)	0.03%<br>(2, 100.0%)	0.0%<br>(0,0.0%)	<.0001
Catheterization	0.97%<br>(440, 100.0%)	1.08%<br>(254, 100.0%)	0.62%<br>(157, 100.0%)	2.12%<br>(86, 100.0%)	<.0001
51701	0.21%<br>(56, 12.73%)	0.18%<br>(30, 11.81%)	0.12%<br>(13, 8.28%)	0.47%<br>(17, 19.77%)	0.0575
51702	0.61%<br>(256, 58.18%)	0.69%<br>(146, 57.48%)	0.42%<br>(120, 76.43%)	1.34%<br>(55, 63.95%)	0.0008
51703	0.11%<br>(34, 7.73%)	0.14%<br>(29, 11.42%)	0.05%<br>(15, 9.55%)	0.31%<br>(14, 16.28%)	0.0507
51102	0.05%<br>(94, 21.36%)	0.07%<br>(49, 19.29%)	0.03%<br>(9, 5.73%)	0.00%<br>(0, 0.00%)	0.4839
Clot Removal	0.41%<br>(90, 100.0%)	0.20%<br>(22, 100.0%)	0.08%<br>(6, 100.0%)	0.23%<br>(3, 100.0%)	<.0001
52001	0.41%<br>(90, 100.0%)	0.20%<br>(22, 100.0%)	0.08%<br>(6, 100.0%)	0.23%<br>(3, 100.0%)	<.0001
Cystoscopy	2.99%<br>(651, 100.0%)	3.50%<br>(386, 100.0%)	4.26%<br>(319, 100.0%)	3.84%<br>(51, 100.0%)	<.0001
52351	0.20%<br>(44, 6.76%)	0.05%<br>(6, 1.55%)	0.05%<br>(4, 1.25%)	0.08%<br>(1, 1.96%)	0.0007
52000	2.79%<br>(607, 93.24%)	3.44%<br>(380, 98.45%)	4.20%<br>(315, 98.75%)	3.77%<br>(50, 98.04%)	<.0001
52005	0.50%<br>(108, 16.59%)	0.28%<br>(31, 8.03%)	0.27%<br>(20, 6.27%)	0.30%<br>(4, 7.84%)	0.0049
52007	0.00%<br>(1, 0.15%)	0.00%<br>(0, 0.00%)	0.00%<br>(0, 0.00%)	0.00%<br>(0, 0.00%)	0.8223
52327	0.00%<br>(0, 0.00%)	0.00%<br>(0, 0.00%)	0.00%<br>(0, 0.00%)	0.00%<br>(0, 0.00%)	1.0000
52204	0.58%<br>(127, 19.51%)	0.35%<br>(39, 10.10%)	0.11%<br>(8, 2.51%)	0.15%<br>(2, 3.92%)	<.0001
Fulgration	0.57%<br>(125, 100.0%)	0.38%<br>(42, 100.0%)	0.53%<br>(40, 100.0%)	0.45%<br>(6, 100.0%)	0.1337
52214	0.29%<br>(63, 50.40%)	0.17%<br>(19, 45.24%)	0.45%<br>(34, 85.00%)	0.30%<br>(4, 66.67%)	0.0064
52224	0.28%<br>(61, 48.80%)	0.21%<br>(23, 54.76%)	0.08%<br>(6, 15.00%)	0.15%<br>(2, 33.33%)	0.0140
52250	0.00%<br>(1, 0.80%)	0.00%<br>(0, 0.00%)	0.00%<br>(0, 0.00%)	0.00%<br>(0, 0.00%)	0.8223
0T5D0ZZ	0.00%<br>(0, 0.00%)	0.00%<br>(0, 0.00%)	0.00%<br>(0, 0.00%)	0.00%<br>(0, 0.00%)	1.0000
0T5D3ZZ	0.00%<br>(0, 0.00%)	0.00%<br>(0, 0.00%)	0.00%<br>(0, 0.00%)	0.00%<br>(0, 0.00%)	1.0000
0T5D4ZZ	0.00%<br>(0, 0.00%)	0.00%<br>(0, 0.00%)	0.00%<br>(0, 0.00%)	0.00%<br>(0, 0.00%)	1.0000
0T5D7ZZ	0.00%<br>(0, 0.00%)	0.00%<br>(0, 0.00%)	0.00%<br>(0, 0.00%)	0.00%<br>(0, 0.00%)	1.0000
0T5D8ZZ	0.00%<br>(0, 0.00%)	0.00%<br>(0, 0.00%)	0.00%<br>(0, 0.00%)	0.00%<br>(0, 0.00%)	1.0000
0T5DXZZ	0.00%<br>(0, 0.00%)	0.00%<br>(0, 0.00%)	0.00%<br>(0, 0.00%)	0.00%<br>(0, 0.00%)	1.0000
Incontinence	0.00%<br>(1, 100.0%)	0.02%<br>(2, 100.0%)	0.03%<br>(2, 100.0%)	0.0%<br>(0,0.0%)	0.4170

	TURP	GreenLight PVP	UroLift PUL	Rezum WVTT	Log Rank p-value
53440	0.00% (0, 0.00%)	0.00% (0, 0.00%)	0.00% (0, 0.00%)	0.0% (0,0.0%)	1.0000
53442	0.00% (0, 0.00%)	0.00% (0, 0.00%)	0.00% (0, 0.00%)	0.0% (0,0.0%)	1.0000
53445	0.00% (1, 100.0%)	0.00% (0, 0.00%)	0.00% (0, 0.00%)	0.0% (0,0.0%)	0.8223
53446	0.00% (0, 0.00%)	0.00% (0, 0.00%)	0.00% (0, 0.00%)	0.0% (0,0.0%)	1.0000
53449	0.00% (0, 0.00%)	0.00% (0, 0.00%)	0.00% (0, 0.00%)	0.0% (0,0.0%)	1.0000
53447	0.00% (0, 0.00%)	0.00% (0, 0.00%)	0.00% (0, 0.00%)	0.0% (0,0.0%)	1.0000
53448	0.00% (0, 0.00%)	0.00% (0, 0.00%)	0.00% (0, 0.00%)	0.0% (0,0.0%)	1.0000
51715	0.00% (1, 100.0%)	0.02% (2, 100.0%)	0.03% (2, 100.0%)	0.0% (0,0.0%)	0.4170
0TUC8JZ	0.00% (0, 0.00%)	0.00% (0, 0.00%)	0.00% (0, 0.00%)	0.0% (0,0.0%)	1.0000
0TUD8JZ	0.00% (0, 0.00%)	0.00% (0, 0.00%)	0.00% (0, 0.00%)	0.0% (0,0.0%)	1.0000
3E0K3GC	0.00% (0, 0.00%)	0.00% (0, 0.00%)	0.00% (0, 0.00%)	0.0% (0,0.0%)	1.0000
3E0K8GC	0.00% (0, 0.00%)	0.00% (0, 0.00%)	0.00% (0, 0.00%)	0.0% (0,0.0%)	1.0000
Incontinence Artificial sphincter	0.0% (0,0.0%)	0.0% (0,0.0%)	0.0% (0,0.0%)	0.0% (0,0.0%)	1.0000
0THC0LZ	0.0% (0,0.0%)	0.0% (0,0.0%)	0.0% (0,0.0%)	0.0% (0,0.0%)	1.0000
0THC3LZ	0.0% (0,0.0%)	0.0% (0,0.0%)	0.0% (0,0.0%)	0.0% (0,0.0%)	1.0000
0THC4LZ	0.0% (0,0.0%)	0.0% (0,0.0%)	0.0% (0,0.0%)	0.0% (0,0.0%)	1.0000
0THC7LZ	0.0% (0,0.0%)	0.0% (0,0.0%)	0.0% (0,0.0%)	0.0% (0,0.0%)	1.0000
0THC8LZ	0.0% (0,0.0%)	0.0% (0,0.0%)	0.0% (0,0.0%)	0.0% (0,0.0%)	1.0000
0THD0LZ	0.0% (0,0.0%)	0.0% (0,0.0%)	0.0% (0,0.0%)	0.0% (0,0.0%)	1.0000
0THD3LZ	0.0% (0,0.0%)	0.0% (0,0.0%)	0.0% (0,0.0%)	0.0% (0,0.0%)	1.0000
0THD4LZ	0.0% (0,0.0%)	0.0% (0,0.0%)	0.0% (0,0.0%)	0.0% (0,0.0%)	1.0000
0THD7LZ	0.0% (0,0.0%)	0.0% (0,0.0%)	0.0% (0,0.0%)	0.0% (0,0.0%)	1.0000
0THD8LZ	0.0% (0,0.0%)	0.0% (0,0.0%)	0.0% (0,0.0%)	0.0% (0,0.0%)	1.0000
0THDXLZ	0.0% (0,0.0%)	0.0% (0,0.0%)	0.0% (0,0.0%)	0.0% (0,0.0%)	1.0000
Infection	0.00% (1, 100.0%)	0.0% (0,0.0%)	0.0% (0,0.0%)	0.0% (0,0.0%)	0.8223
55720	0.00% (1, 100.0%)	0.0% (0,0.0%)	0.0% (0,0.0%)	0.0% (0,0.0%)	0.8223

	TURP	GreenLight PVP	UroLift PUL	Rezum WVTT	Log Rank p-value
55725	0.00% (0, 0.00%)	0.0% (0,0.0%)	0.0% (0,0.0%)	0.0% (0,0.0%)	1.0000
Nephrostomy	0.00% (1, 100.0%)	0.0% (0,0.0%)	0.0% (0,0.0%)	0.0% (0,0.0%)	0.8223
50432	0.00% (1, 100.0%)	0.0% (0,0.0%)	0.0% (0,0.0%)	0.0% (0,0.0%)	0.8223
52334	0.00% (0, 0.00%)	0.0% (0,0.0%)	0.0% (0,0.0%)	0.0% (0,0.0%)	1.0000
0V9000Z	0.00% (0, 0.00%)	0.0% (0,0.0%)	0.0% (0,0.0%)	0.0% (0,0.0%)	1.0000
0V900ZX	0.00% (0, 0.00%)	0.0% (0,0.0%)	0.0% (0,0.0%)	0.0% (0,0.0%)	1.0000
0V900ZZ	0.00% (0, 0.00%)	0.0% (0,0.0%)	0.0% (0,0.0%)	0.0% (0,0.0%)	1.0000
0V9030Z	0.00% (0, 0.00%)	0.0% (0,0.0%)	0.0% (0,0.0%)	0.0% (0,0.0%)	1.0000
0V903ZX	0.00% (0, 0.00%)	0.0% (0,0.0%)	0.0% (0,0.0%)	0.0% (0,0.0%)	1.0000
0V903ZZ	0.00% (0, 0.00%)	0.0% (0,0.0%)	0.0% (0,0.0%)	0.0% (0,0.0%)	1.0000
0V9040Z	0.00% (0, 0.00%)	0.0% (0,0.0%)	0.0% (0,0.0%)	0.0% (0,0.0%)	1.0000
0V904ZX	0.00% (0, 0.00%)	0.0% (0,0.0%)	0.0% (0,0.0%)	0.0% (0,0.0%)	1.0000
0V904ZZ	0.00% (0, 0.00%)	0.0% (0,0.0%)	0.0% (0,0.0%)	0.0% (0,0.0%)	1.0000
0V9070Z	0.00% (0, 0.00%)	0.0% (0,0.0%)	0.0% (0,0.0%)	0.0% (0,0.0%)	1.0000
0V907ZX	0.00% (0, 0.00%)	0.0% (0,0.0%)	0.0% (0,0.0%)	0.0% (0,0.0%)	1.0000
0V907ZZ	0.00% (0, 0.00%)	0.0% (0,0.0%)	0.0% (0,0.0%)	0.0% (0,0.0%)	1.0000
0V9080Z	0.00% (0, 0.00%)	0.0% (0,0.0%)	0.0% (0,0.0%)	0.0% (0,0.0%)	1.0000
0V908ZX	0.00% (0, 0.00%)	0.0% (0,0.0%)	0.0% (0,0.0%)	0.0% (0,0.0%)	1.0000
0V908ZZ	0.00% (0, 0.00%)	0.0% (0,0.0%)	0.0% (0,0.0%)	0.0% (0,0.0%)	1.0000
Stone Removal	0.35% (76, 100.0%)	0.34% (38, 100.0%)	0.17% (13, 100.0%)	0.68% (9, 100.0%)	0.0123
51050	0.00% (0, 0.00%)	0.00% (0, 0.00%)	0.00% (0, 0.00%)	0.00% (0, 0.00%)	1.0000
52310	0.15% (33, 43.42%)	0.10% (11, 28.95%)	0.11% (8, 61.54%)	0.68% (9, 100.0%)	<.0001
52315	0.00% (1, 1.32%)	0.02% (2, 5.26%)	0.07% (5, 38.46%)	0.00% (0, 0.00%)	0.0094
52317	0.11% (23, 30.26%)	0.15% (17, 44.74%)	0.00% (0, 0.00%)	0.00% (0, 0.00%)	0.0057
52318	0.09% (19, 25.00%)	0.07% (8, 21.05%)	0.00% (0, 0.00%)	0.00% (0, 0.00%)	0.0570
Transfusion	0.0% (0,0.0%)	0.0% (0,0.0%)	0.0% (0,0.0%)	0.0% (0,0.0%)	1.0000

	TURP	GreenLight PVP	UroLift PUL	Rezum WVTT	Log Rank p-value
36430	0.0% (0,0.0%)	0.0% (0,0.0%)	0.0% (0,0.0%)	0.0% (0,0.0%)	1.0000
Ureteral Reimplantation	0.0% (0,0.0%)	0.0% (0,0.0%)	0.0% (0,0.0%)	0.0% (0,0.0%)	1.0000
50947	0.0% (0,0.0%)	0.0% (0,0.0%)	0.0% (0,0.0%)	0.0% (0,0.0%)	1.0000
50948	0.0% (0,0.0%)	0.0% (0,0.0%)	0.0% (0,0.0%)	0.0% (0,0.0%)	1.0000
Urethral Stent Placement (Spanner)	0.0% (0,0.0%)	0.0% (0,0.0%)	0.04% (3, 100.0%)	0.45% (6, 100.0%)	<.0001
53855	0.0% (0,0.0%)	0.0% (0,0.0%)	0.00% (0, 0.00%)	0.45% (6, 100.0%)	<.0001
52282	0.0% (0,0.0%)	0.0% (0,0.0%)	0.04% (3, 100.0%)	0.00% (0, 0.00%)	0.0034
Urethral Stricture	1.14% (249, 100.0%)	0.69% (76, 100.0%)	0.37% (28, 100.0%)	0.30% (4, 100.0%)	<.0001
52275	0.00% (0, 0.00%)	0.00% (0, 0.00%)	0.00% (0, 0.00%)	0.00% (0, 0.00%)	1.0000
52277	0.00% (0, 0.00%)	0.00% (0, 0.00%)	0.00% (0, 0.00%)	0.00% (0, 0.00%)	1.0000
0T7D0DZ	0.00% (0, 0.00%)	0.00% (0, 0.00%)	0.00% (0, 0.00%)	0.00% (0, 0.00%)	1.0000
0T7D0ZZ	0.00% (0, 0.00%)	0.00% (0, 0.00%)	0.00% (0, 0.00%)	0.00% (0, 0.00%)	1.0000
0T7D3DZ	0.00% (0, 0.00%)	0.00% (0, 0.00%)	0.00% (0, 0.00%)	0.00% (0, 0.00%)	1.0000
0T7D3ZZ	0.00% (0, 0.00%)	0.00% (0, 0.00%)	0.00% (0, 0.00%)	0.00% (0, 0.00%)	1.0000
0T7D4DZ	0.00% (0, 0.00%)	0.00% (0, 0.00%)	0.00% (0, 0.00%)	0.00% (0, 0.00%)	1.0000
0T7D4ZZ	0.00% (0, 0.00%)	0.00% (0, 0.00%)	0.00% (0, 0.00%)	0.00% (0, 0.00%)	1.0000
0T7D7DZ	0.00% (0, 0.00%)	0.00% (0, 0.00%)	0.00% (0, 0.00%)	0.00% (0, 0.00%)	1.0000
0T7D7ZZ	0.00% (0, 0.00%)	0.00% (0, 0.00%)	0.00% (0, 0.00%)	0.00% (0, 0.00%)	1.0000
0T7D8DZ	0.00% (0, 0.00%)	0.00% (0, 0.00%)	0.00% (0, 0.00%)	0.00% (0, 0.00%)	1.0000
0T7D8ZZ	0.00% (0, 0.00%)	0.00% (0, 0.00%)	0.00% (0, 0.00%)	0.00% (0, 0.00%)	1.0000
0TQD0ZZ	0.00% (0, 0.00%)	0.00% (0, 0.00%)	0.00% (0, 0.00%)	0.00% (0, 0.00%)	1.0000
0TQD3ZZ	0.00% (0, 0.00%)	0.00% (0, 0.00%)	0.00% (0, 0.00%)	0.00% (0, 0.00%)	1.0000
0TQD4ZZ	0.00% (0, 0.00%)	0.00% (0, 0.00%)	0.00% (0, 0.00%)	0.00% (0, 0.00%)	1.0000
0TQD7ZZ	0.00% (0, 0.00%)	0.00% (0, 0.00%)	0.00% (0, 0.00%)	0.00% (0, 0.00%)	1.0000
0TQD8ZZ	0.00% (0, 0.00%)	0.00% (0, 0.00%)	0.00% (0, 0.00%)	0.00% (0, 0.00%)	1.0000
0TQDXZZ	0.00% (0, 0.00%)	0.00% (0, 0.00%)	0.00% (0, 0.00%)	0.00% (0, 0.00%)	1.0000

	TURP	GreenLight PVP	UroLift PUL	Rezum WVTT	Log Rank p-value
53415	0.00%<br>(0, 0.00%)	0.00%<br>(0, 0.00%)	0.00%<br>(0, 0.00%)	0.00%<br>(0, 0.00%)	1.0000
53420	0.00%<br>(0, 0.00%)	0.00%<br>(0, 0.00%)	0.00%<br>(0, 0.00%)	0.00%<br>(0, 0.00%)	1.0000
53425	0.00%<br>(0, 0.00%)	0.00%<br>(0, 0.00%)	0.00%<br>(0, 0.00%)	0.00%<br>(0, 0.00%)	1.0000
53020	0.00%<br>(1, 0.40%)	0.01%<br>(1, 1.32%)	0.00%<br>(0, 0.00%)	0.00%<br>(0, 0.00%)	0.8394
53510	0.00%<br>(0, 0.00%)	0.00%<br>(0, 0.00%)	0.00%<br>(0, 0.00%)	0.00%<br>(0, 0.00%)	1.0000
53515	0.00%<br>(0, 0.00%)	0.00%<br>(0, 0.00%)	0.00%<br>(0, 0.00%)	0.00%<br>(0, 0.00%)	1.0000
52283	0.03%<br>(6, 2.41%)	0.02%<br>(2, 2.63%)	0.00%<br>(0, 0.00%)	0.00%<br>(0, 0.00%)	0.4763
52276	0.30%<br>(65, 26.10%)	0.05%<br>(5, 6.58%)	0.13%<br>(10, 35.71%)	0.00%<br>(0, 0.00%)	<.0001
52281	0.46%<br>(100, 40.16%)	0.42%<br>(46, 60.53%)	0.17%<br>(13, 46.43%)	0.30%<br>(4, 100.0%)	0.0068
53600	0.17%<br>(36, 14.46%)	0.08%<br>(9, 11.84%)	0.03%<br>(2, 7.14%)	0.00%<br>(0, 0.00%)	0.0052
53601	0.04%<br>(9, 3.61%)	0.03%<br>(3, 3.95%)	0.03%<br>(2, 7.14%)	0.00%<br>(0, 0.00%)	0.7822
53605	0.03%<br>(6, 2.41%)	0.00%<br>(0, 0.00%)	0.01%<br>(1, 3.57%)	0.00%<br>(0, 0.00%)	0.3040
53620	0.04%<br>(9, 3.61%)	0.03%<br>(3, 3.95%)	0.00%<br>(0, 0.00%)	0.00%<br>(0, 0.00%)	0.2907
53621	0.00%<br>(0, 0.00%)	0.00%<br>(0, 0.00%)	0.00%<br>(0, 0.00%)	0.00%<br>(0, 0.00%)	1.0000
52341	0.03%<br>(7, 2.81%)	0.03%<br>(3, 3.95%)	0.00%<br>(0, 0.00%)	0.00%<br>(0, 0.00%)	0.4287
52342	0.00%<br>(0, 0.00%)	0.00%<br>(0, 0.00%)	0.00%<br>(0, 0.00%)	0.00%<br>(0, 0.00%)	1.0000
52344	0.04%<br>(8, 3.21%)	0.05%<br>(5, 6.58%)	0.00%<br>(0, 0.00%)	0.00%<br>(0, 0.00%)	0.2993
52345	0.00%<br>(1, 0.40%)	0.00%<br>(0, 0.00%)	0.00%<br>(0, 0.00%)	0.00%<br>(0, 0.00%)	0.8223
52346	0.01%<br>(2, 0.80%)	0.00%<br>(0, 0.00%)	0.00%<br>(0, 0.00%)	0.00%<br>(0, 0.00%)	0.6092
